# Potential fungicidal and antiaflatoxigenic effects of cinnamon essential oils on *Aspergillus flavus* inhabiting the stored wheat grains

**DOI:** 10.1186/s12870-024-05065-w

**Published:** 2024-05-13

**Authors:** Manar M. Abdel Gwad, Ashraf S. A. El-Sayed, Gamal M. Abdel-Fattah, Mohamed Abdelmoteleb, Ghada G. Abdel-Fattah

**Affiliations:** 1 Enzymology and Fungal Biotechnology Lab, Botany and Microbiology Department, Faculty of Science, Zagazig University, Zagazig, 44519 Egypt; 2 Botany Department, Faculty of Science, Mansoura University, Mansoura, Egypt

**Keywords:** Aflatoxins, *Aspergillus flavus*, Wheat grains, Solid state fermentation, SEM analysis, Molecular expression

## Abstract

Wheat is one of the essential crops for the human and animal nutrition, however, contamination with aflatoxigenic fungi, due to the improper storage conditions and high humidity, was the main global threats. So, preventing the growth of aflatoxigenic fungi in stored wheat grains, by using different essential oils was the main objective of this work. *Aspergillus flavus* EFBL-MU12 PP087400, EFBL-MU23 PP087401 and EFBL-MU36 PP087403 isolates were the most potent aflatoxins producers inhabiting wheat grains. The effect of storage conditions of wheat grains “humidity, temperature, incubation period, and pH” on growth of *A. flavus*, was assessed by the response surface methodology using Plackett-Burman design and FCCD. The highest yield of aflatoxins EFBL-MU12 B_1_ and B_2_ by *A. flavus* grown on wheat grains were 145.3 and 7.6 μg/kg, respectively, at incubation temperature 35°C, 16% moisture contents, initial pH 5.0, and incubated for 14 days. The tested oils had a powerful antifungal activity for the growth and aflatoxins production by *A. flavus* in a concentration-dependent manner. Among these oils, cinnamon oil had the highest fungicidal activity for *A. flavus* at 0.125%, with about 85-90 % reduction to the aflatoxins B_1_ and B_2_, conidial pigmentation and chitin contents on wheat grains. From the SEM analysis, cinnamon oils had the most deleterious effect on *A. flavus* with morphological aberrations to the conidial heads, vegetative mycelia, alteration in conidiophores identity, hyphae shrank, and winding. To emphasize the effect of the essential oils on the aflatoxins producing potency of *A. flavus,* the molecular expression of the aflatoxins biosynthetic genes was estimated by RT-qPCR. The molecular expression of *nor-1, afLR*, *pKsA* and *afLJ* genes was suppressed by 94-96%, due to cinnamon oil at 0.062% compared to the control. Conclusively, from the results, cinnamon oils followed by the peppermint oils displayed the most fungicidal activity for the growth and aflatoxins production by *A. flavus* grown on wheat grains.

**Supplementary Information:**

The online version contains supplementary material available at 10.1186/s12870-024-05065-w.

## Introduction

Wheat is one of the third most important cereals worldwide, after maize and rice [1], representing an important strategic material for human nutrition by manufacturing flour [2], by producing a strong cohesive dough, that is essential in a wide range of food products [3]. Cultivation, harvesting, drying, and storage are the man critical steps in wheat preparation, however, poor harvesting practices, and improper storage could raise the chance of microbial contamination and infection [4], affecting the quality and safety of wheat and its byproducts play a significant role in protecting the human health [5]. Storage of wheat grains to fulfill human needs, domestic cereal industries and animal needs is a major challenge especially in developing countries. Stored grains ecosystem is as the habitat for various species of heterotrophic fungi and bacteria. Due to the improper storage conditions of wheat grains in the developing countries, fungi are the major cause of loss on long term storage periods with the lack of efficient control of temperature and moisture contents [6, 7]. With the long term storage, and higher moisture contents and temperature, the natural molds inocula started to develop and cause an obvious deterioration [8]. Fungal deterioration of stored grains is a common problem especially in tropical hot and humid climate like Egypt. The contamination of stored grains by various fungi leads to a decline in crop yield, reduction in quality, significant economic losses as well as makes a possible hazard for human health because of the production of mycotoxins by the fungi [9]. Mycotoxins are low molecular weight, that usually produced in the field and during the storage of agricultural products by certain fungi such as *Aspergillus*, *Fusarium*, and *Alternaria* spp, with a deleterious effect on human health and animals, causing an acute and chronic diseases [10, 11]. The major mycotoxins occurs in food and feed are aflatoxins, ochratoxins, trichothecenes, zearalenone and fumonisins [12]. Aflatoxins (AFs) are mainly produced by toxigenic strains of *Aspergillus flavus* and *A. parasiticus* as a contaminants of food and agricultural products [13], that belongs to the section Flavi. This section includes several economically important species mainly *A. oryzae* and *A. sojae* that have been used various in food fermentation industries, without aflatoxin producing potency. There are about 20 different types of aflatoxins, the four major ones are aflatoxins B_1_, B_2_, G_1_ & G_2_, among them, aflatoxin B1 was the most potent one associated with human toxicity and carcinogenicity [14], as classified as Group I carcinogen by FAO and WHO [15]. So, many countries constrain a stringent limit to aflatoxins, especially to B_1_ type in cereals. For example, the European Union limits the total amount of aflatoxins in cereals should to be not exceed than 4.0 μg/kg [16]. So, the prevention of fungal growth on agricultural commodities to prevent aflatoxin contamination is of utmost importance in evaluating food safety. Several approaches have been considered for the management of aflatoxigenic fungi via pre-harvest strategies and post-harvest practices which include multiple physical, chemical and biological methods [17]. The chemical and physical approaches are so expensive with potential residual toxicity [18], while the biological methods utilize microorganisms and their enzymes for decontaminating mycotoxins in food [19], often resulting undesirable products. So, searching for an eco-friendly and sustainable methods to combat fungal colonization and aflatoxins production in food with no aflatoxin residues [20] ‘green image’ foods [21].

Plant extracts essential oils (EOs) with their constituents of aromatic, volatile, oily liquids could be an alternative approach for control of the aflatoxigenic fungi [22]. EOs can interrupt the plasma membrane structure, causing damage to the cellular organelles of the toxigenic fungi [23]. Recently, several reports explore the potential antifungal activity of essential oils against *Aspergillus* and *Fusarium* on cereals [24–26]. However, limited data about the effect of EOs on the inhibition of *Aspergillus* growth and aflatoxin biosynthesis were documented. Thus, this study aimed to assess the potency of different commercial essential oils in controlling the growth and aflatoxins production by aflatoxigenic fungi of the stored wheat grains under submerged and solid state-fermentation processes.

## Materials and methods

### Collection of samples

Thirty samples of wheat grains were gathered from different local markets in Mansoura city, Dakahlia governorate, Egypt, from December 2021 to April, 2022, that were marketed for human consumption. The samples were collected according to the guidelines of the European Commission and the International Organization for Standardization [27]. Each aggregate sample is of 1 kg in total weight, 3 replicates were taken for each sample, mixed to prepare one composite sample. Each sample was placed in a sealed sterile polyethylene bag, and surveyed for the presence of fungi and the rest of the samples were kept frozen at -20°C for further analysis [28].

### Mycological analysis

#### Isolation of the mycoflora from the collected wheat samples

The number of fungal colonies was determined by plating 0.5 gram (10 grains per plate) of each sample randomly to the plates of sterilized potato dextrose agar medium (PDA) (200 g potato extracts, 20 g dextrose, 20 g agar and 1 L distilled water) of 0.5 mg chloramphenicol /L [29]. Triplicates of the plates were incubated at 28±2°C for 7 days, the developed fungal colonies were purified on Czapek’s-Dox agar medium (Rapper and Fennel, 1965) and potato dextrose agar medium (PDA), and kept at 4°C until for uses. The fungal colonies were counted per gram of each sample. To select species, belonging to the genera *Aspergillus* and *Penicillium* and to decrease the spreading of fast growing fungi (almost phycomycetes), a sterile solution of 8% NaCl was added to the medium to adjust the water activity [30].

#### Morphological and Molecular Identification of fungal isolates

The recovered fungal colonies were subcultured into Czapek’s-Dox agar medium for identification based on their morphological characteristics, sporulation and colony color [31]. The fungal genera were identified according to their microscopical and macroscopical features of the universally keys [32–35]. The potent isolates were molecularly identified based on the sequence of their internal transcribed spacers (ITS) using genomic DNA as template for PCR [36–38], with primers ITS4 5′-GGAAGTAAAAGTCGTAACAAGG-3′ and ITS5 5′-TCCTCCG CTTATTGATATGC-3′. The reaction of PCR contains 10 μl of 2 × PCR master mixture (Cat. # 25027), 1 μl of gDNA, 1 μl of primers (10 pmol) in a 20 μl total volume. The PCR program was initial denaturation at 94 °C for 2 min, 35 cycles at 94 °C for 30 s, 55 °C for 10 s, 72 °C for 30 s, and final extension at 72 °C for 2 min. The PCR products were analyzed by 1.5 % agarose gel, sequenced by Applied Biosystems Sequencer with the same primer set. The ITS sequences were non-redundantly BLAST searched on NCBI database, aligned with ClustalW muscle algorithm by MEGA 10 software [39, 40], and the phylogenetic analysis was done by neighbor-joining method [41].

#### Preliminary detection of aflatoxigenic fungi in the plate cultures by fluorescence analysis

For determination of aflatoxins producing potency, the isolates of *Aspergillus* species were cultivated on PDA at 25°C for 7 days, by placing a mycelial plug centrally on Czapek’s-Dox agar medium [42]. The plates were incubated at 25ºC for 4 days in the dark, then the potency of aflatoxins producing potency was assessed by illuminating the plates with ultraviolet (UV) light at λ_365_ nm, and the positive/ negative emission of fluorescence was recorded [43].

#### Extraction and quantification of aflatoxins from the submerged and solid-state fermented fungal cultures

The aflatoxins productivity by the submerged fungal isolates was assayed [44]. Briefly, the tested fungal isolates were cultured in 250 ml Erlenmeyer flasks containing 100 ml yeast extract-sucrose (YES) broth medium (150 g sucrose, 19 g yeast extract, 1g peptone dissolved in 1 L distilled water) [45]. After autoclaving, the flasks were inoculated with 1 ml (10^3^ spores /ml) /50 ml medium per 250 ml Erlenmeyer conical flask, incubated in a static condition at 28°C for 10 days. The fungal cultures were filtered, the fungal biomass was assessed, and the filtrates were used for aflatoxins extraction and quantification. The aflatoxins were extracted from the cultural filtrates by chloroform at 1/1 v/v, the mixtures were shacked for 30 min, then the two layers were separated by separating funnel, and the chloroform layer was filtered over anhydrous sodium sulfate in 250 ml, and evaporated in a water bath (70-80°C) till dryness. The dried residues were dissolved in 1 ml chloroform and kept at -20°C, until analysis.

Twenty-five grams of wheat grains were transferred to 250 ml Erlenmeyer flasks followed by maintenance of moisture content by adding the necessarily required volume of sterilized distilled water [46] to get the desired concentration. The tested essential oils were dissolved in Tween 20 and added to the medium at final concentrations 0.0625, 0.125, 0.25, 0.5, 1, 2 and 4 % v/w. The flasks were then sterilized by autoclave at 121ºC for 20 min. Untreated flasks were used as control. The flasks were inoculated with 1ml of spore suspension of the selected isolates. Spores of isolates were prepared by growing the fungi on PDA slant for 7 days and harvested using sterile distilled water and used as the inoculum for the fermentative production of aflatoxins. The flasks were closed tightly and distributed as evenly as possible by shaking vigorously and then incubated at 28±2°C for 10 days. Aflatoxins were extracted from the wheat grains [47]. The aflatoxins of the solid state fermented fungal cultures were estimated. Briefly, twenty-five grams of the homogenized sample was amended with 125 ml methanol/water (55 /45 v/v), 100 ml hexane and 2 g sodium chloride were added. The mixture was vigorously shaken for 30 min on an orbital shaker, the mixture was filtered, and the filtrate was stand for 30 min. The lower aqueous methanol phase (25 ml) was taken, chloroform was added, and shacked for 5 min, standing for 10 min. The layer of chloroform was dried over anhydrous sodium sulfate, and the chloroform was evaporated at 60°C to dryness.

#### Aflatoxins detection and quantification

The aflatoxins were fractionated by thin layer chromatography (TLC) [48] with slight modifications. Briefly, the extract was spotted on TLC plate, running by solvent system toluene: ethyl acetate: formic acid (6:3:1), in presence of the authentic aflatoxins (B_1_, B_2_, G_1_ and G_2_) [49]. The TLC plates were visualized by UV-illumination at λ_360_ nm, each type of toxin distinguished by fluorescence properties. The blue fluorescence emission refers to aflatoxins B_1_ and B_2,_ while the green fluorescence refers to G_1_ and G_2_. The intensity of the putative spots corresponding to the rate of mobility and color of the authentic one was calculated by the Image J software package (El-Sayed et al., 2019, 2020, 2022), regarding to the known concentration of each authentic toxin. The positive aflatoxins samples on TLC were further checked for their purity and concentration by HPLC system (YOUNG In, Chromass, 9110+ Quaternary Pump, Korea) of RP-C18 column (Eclipse Plus C18 4.6 mm × 150 mm, Cat. #959963-902) with fluorescence detector. The extracted aflatoxins were derivatized with trifluoroacetic acid. The mobile phase was Water: Acetonitrile: Methanol (65: 5: 30) at a flow rate 1.0 ml/min. The injection sample was 20 μl. The fluorescence wavelength for detection was at excitation λ_365_ nm and emission λ_440_ nm. Standard of aflatoxins B1, B2, G1 and G2 were used.

#### Nutritional optimization of the potent isolate for maximizing the aflatoxin productivity by the Plackett-Burman design

The effect of cultural conditions including temperature, moisture content, pH and incubation time on aflatoxin B_1_, B_2_, G_1_, and G_2_ production in wheat grains inoculated with the potent isolate were studied by Plackett-Burman design [50]. Four parameters were represented by high (+1) and low (-1) levels. The first ordered polynomial model equation (Eq.1) was calculated from the coefficient of determination (R2), and F-test. The significant effects were assessed from Eq. 2, the significant factors were validated, and the model accuracy was calculated (Eq. 3).

1 $${Y =\beta }_{0}+\sum { \beta }_{i}{\mathrm{\rm X}}_{i},$$

Y is the aflatoxin yield (μg/kg), β_0_ is the model intercept, β_i_ is the factor estimate and X_i_ represents the factor.

2 $$\mathrm{Main effects }=\sum \left(+1\right)/n\left(+1\right)-\sum \frac{-1}{n\left(-1\right)},$$

3 $$\mathrm{Model accuracy }=\frac{{\text{Y}}{ }_{{\text{Experiment}}}}{{\mathrm{Y }}_{{\text{Calculated}}}} \times 100,$$

The most influential variables derived from the Plackett-Burman Design (PBD), influencing on the aflatoxins production by the fungus were optimized by the Central Composite Design (CCD) [51, 52]. A second-order polynomial model was used for predicting the optimum storage conditions for aflatoxins production (Eq. 4):

4 $${Y =\beta }_{0}+\sum {\beta }_{i}{\mathrm{\rm X}}_{i}+\sum {\beta }_{\begin{array}{c}ii\\ \end{array}}{x}_{ii}+\sum {\beta }_{ij}{\mathrm{\rm X}}_{ij},$$

β_i_ is the variables regression coefficient, β_ii_ is the regression coefficient of square effects, and β_ij_ is the regression coefficient of the interactions.

#### Effect of plant essential oils on growth, melanin pigment and chitin content of the potent fungal isolate

Four purified cinnamon oil *(Cinnamomum zeylanicum)*, clove oil *(Syzygium aromaticum)*, garlic oil *(Allium sativum),* and pepper mint oil *(Mentha peperita)* were obtained from Badawyia Company, Mansoura, Egypt. The activity of the tested essential oils on the growth of the aflatoxigenic fungus was performed on PDA media [53] amended with the different concentrations of the oils (0.0625, 0.125, 0.25, 0.5, 1, 2 and 4 % v/v). The sterilized melted media was amended with the different corresponding concentrations of the tested oils in the presence of 100 µl Tween 20 per 100 ml medium for enhancing the oil solubility. The PDA plates were centrally inoculated with a disc of 6 days PDA-culture of the selected fungal isolate. Triplicates of each treatment were conducted. Additionally, positive controls (without oil) were prepared by the same procedure. The plate cultures were incubated at 28±2°C and the colony diameter was daily measured until control petri dishes were fully covered with mycelia. The concentration of essential oil which completely inhibited the fungal growth was designated as minimum inhibitory concentration (MIC). The percentage of Mycelial Growth Inhibition (MGI) was calculated according to the equation suggested by [54] as follows:

$$\mathrm{Mycelial Growth Inhibition }({\text{MGI}})\mathrm{ \% }=({\text{Dc}}-{\text{Dt}})/\mathrm{Dc }\times 100$$

where Dc (cm) is colony diameter of control sets and Dt (cm) is colony diameter measured in treatment sets.

The effect of essential oils on melanin pigmentation of the selected fungi was assessed [55]. Briefly, 0.5 g of the fungal biomass was pulverized in 2 M NaOH (pH 10.5), incubated for 48 h, centrifuged at 5000 rpm for 20 min, the supernatant was acidified to 2.5 with 2 M HCl. The mixture was incubated for 12 h, centrifuged at 5000 rpm, the supernatant was decanted, and the precipitate was collected, and hydrolyzed with 6 M HCl at 100°C for 2h. The mixture was amended with ethylacetate, the precipitate was dissolved in 2M NaOH, centrifuged at 5000 rpm for 15 min, and the supernatant was collected, acidified with 6 M HCl, then further dried. The purified melanin was dissolved in 1 mL borate buffer (pH 8.0, 100mM) and measured at wavelength λ_459_ nm [56], compared to the authentic melanin.

The fungal growth on wheat grains was determined in response to chitin contents after hydrolysis into N-acetyl glucosamine according to [57], with slight modifications. Briefly, 2 g of the fungal biomass was amended with5 ml of 72% H_2_SO_4_, agitated for 30 min, diluted by adding 54 ml distilled water and autoclaved for 2 h. The hydrolyzate was neutralized (pH 7.0) by 10 N NaOH, the glucosamine contents were quantified colorimetrically [58]. An aliquot of 3 mL of hydrolyzate was added to an equal volume of 5% (w/v) NaNO_2_ and 5% (w/v) KHSO_4_, agitated for 15 min, centrifuged at 1500 × g, for 2 min, and then 3 mL of the supernatant was amended by 1 ml 12.5% (w/v) NH_4_SO_3_NH_2,_ and agitated for 5 min. One ml of freshly prepared 0.5% (w/v) 3-methyl-2-benzothiazolone hydrazone hydrochloride was added, boiling water bath for 3 min, cooling, then 1 ml of freshly prepared 0.5% (w/v) FeCl_3_ was added, the absorbance was measured at λ_650_ nm. Control medium of fungus without essential oil, and wheat grain medium without fungus were used as controls.

#### Scanning Electron Microscope (SEM) analysis

The morphological aberrations of the selected fungal isolate in response to the tested essential oils were studied by SEM [59]. The isolate was grown on PDA with the different concentrations of the essential oils, incubated at 28±2°C for 5 days, and then a segment of 5-10 mm of homogenous growth was excised from cultures and prepared for the SEM analysis. The sample was primarily fixed with 2.5% glutaraldehyde solution overnight at 4°C, washed with 0.1 M sodium phosphate buffer solution (pH 7.2), followed by dehydration in ethanol series (30- 95%), for 20 min, and with absolute ethanol for 45 min. The samples were mounted on silver stub and gold covered by cathodic spraying (Polaron gold). The morphological features of the fungus were visualized by scanning electron microscope (JEOL JSM 6510 lv) at 20.00 kv.

#### Molecular expression analysis of aflatoxin biosynthetic genes of the potent fungus with the essential oils treatments

The effect of tested plant essential oils on the biosynthetic potency of aflatoxins by the selected fungus was assessed from the molecular expression of the rate-limiting genes controlling aflatoxins biosynthesis. Different concentrations of oils were added to yeast extract sucrose broth medium inoculated with a plug of fungal inoculum [60]. Cultures with zero oils were used as negative controls. The molecular expression of the aflatoxin regulatory (*aflR*), aflatoxin J (*aflJ*), in addition to the structural genes, norsolonic acid-1 (*nor-1*) and polyketide synthase acetate (*pksA*) were assessed by RT-qPCR analysis. The primer sets were *aflR* 5′-CAACTCGGCGACCATCAGAG-3′, 5′-GGGAAGAGGTGGGTCAGTGT-3′, *aflJ* 5′-ATAAA GTCAGCGGCGTGGT G-3′, 5′-ATGACCGGCACCTTAGCAGT-3′, *nor-1* 5′-GGGATAGAC CGCCTGAGGAG-3′, 5′-CTTCAGCGACGGTTAGTGCC-3′, *pKsA* 5′-TTCTGCATGGGTTC CTTGGC-3′, 5′-CCATTGTGGGCCGGTAAACA-3′ and *β-Actin* gene 5'-ACGGTAT TTCCAA CTGGGACG-3', 5'-TGGAGCTTCGGTCAACAAAACTGG-3'. After incubation for 5 days, the fungal mycelia were collected and subsequently flash frozen with liquid nitrogen, ground to a fine powder and the total RNA was extracted using Total RNA Isolation Kit (Cat#. NA020-0100, GeneDirex Inc, USA), and reverse transcribed to cDNA by GScript First-Strand Synthesis Kit (GeneDirex, USA) with oligo-dT primes. The RT-qPCR reaction mixtures contain cDNA, primers, and Xpert Fast SYBER Green Mastermix (Grisp, Portugal) using the real-time PCR machine, with a program initial denaturation at 95 °C for 3 min, followed by 40 cycles of 95°C for 10 s, 55°C for 15 s, 72°C for 20 s. Melting curve analyses were performed at 72-95 °C. The results were normalized to the house-keeping gene *β-Actin*, as endogenous control, and the expression folds of the target genes were calculated using the Rotor-Gene software 1.7.94, based on 2−∆∆^ct^ formula [61].

#### GC-MS metabolic profiling of the solid-state fermented fungal cultures in response to essential oils

The metabolic profiling of the aflatoxigenic fungal isolate in response to different essential oils was explored from the GC-MS/MS (Agilent Technol) with a mass-selective detector (MSD, Agilent 7000), of polar Agilent HP-5ms (5%-phenylmethyl poly siloxane) column (30 m x 0.25 mm) [62]. The injected sample (1 μl) with helium as carrier gas, velocity 1 ml/min, with the injector and detector temperature were 200°C and 250°C. The parameters were ionization potential 70eV, interface temperature 250°C and acquisition mass range 50-800. The identity of the bioactive compounds in the extracts was determined corresponding to their mass spectra and retention time using NIST library.

#### Fungal deposition

The current isolates of *Aspergillus flavus* EFBL-MU12, EFBL-MU23 and EFBL-MU36 were deposited at genbank with accession number PP087400, PP087401, and PP087403, respectively.

### Statistical analysis

The statistical analyses were performed by CoHort/ CoStat software version 6.4. The data were represented by means and standard deviation (SD). The least significant differences (LSD) were represented by the means at p≤0.05, with ANOVA (analysis of variance).

## Results

### Occurrence of Aflatoxins in wheat grains and isolation of the aflatoxigenic fungi

Thirty samples of wheat grains were collected from different local stores in Mansoura city, Egypt, from December 2021 to April 2022, and used for the determination of their intrinsic aflatoxins contents prior to isolation of their aflatoxigenic fungi. The aflatoxins contents of B_1_, B_2_, G_1_ and G_2_ were determined in the tested wheat grains by the TLC and HPLC. From the thirty tested samples of wheat grains, 23 samples have aflatoxins with fluctuated amounts regarding to the toxins types. From the results (Table 1), the wheat grain sample # 7, displayed the highest aflatoxins contents B_1_ (416.1 μg/kg), B_2_ (0.87 μg/kg), G_1_ (5.1 μg/kg) and G_2_ (95.8 μg/kg), followed by sample #13 that contain 261.4, 1.1, 2.9 and 68.5 μg/kg for aflatoxins B_1_, B_2_, G_1_ and G_2_, respectively. The fluctuation of the aflatoxins contents of the wheat grains samples might be attributed to the improper storage conditions, and higher humidity of the positive aflatoxins samples, which allowing to the growth of aflatoxigenic fungi. Practically, for the positive toxins samples, the aflatoxin B_1_ and G_2_ were the highest concentrations than the B_2_ and G_1_ toxins, among all the positive samples. The negative aflatoxins samples of wheat grains could be the due to proper storage conditions of dryness, thus preventing the growth of the aflatoxigenic fungi, unlike to the positive aflatoxins containing samples.

**Table 1:** Aflatoxins occurrence in stored wheat grain samples

**Sample No.**	**Aflatoxins concentrations (μg/kg)**				**Total amount of aflatoxins ( (μg/kg)**
	**B**_**1**_	**B**_**2**_	**G**_**1**_	**G**_**2**_	
**1**	**76.97**	**1.08**	**5.64**	**13.41**	**97.1**
**2**	**-**	**7.66**	**-**	**20.41**	**28.07**
**3**	**-**	**2.03**	**128.81**	**14.27**	**145.11**
**4**	**-**	**0.168**	**48.43**	**6.36**	**54.958**
**5**	**121.39**	**2.21**	**12.02**	**5.88**	**141.5**
**6**	**-**	**3.05**	**5.68**	**22.26**	**30.99**
**7**	**416.02**	**0.873**	**5.11**	**95.79**	**517.793**
**8**	**-**	**1.75**	**4.38**	**40.81**	**46.94**
**9**	**-**	**2.54**	**-**	**22.33**	**24.87**
**10**	**-**	**-**	**-**	**-**	**0**
**11**	**-**	**-**	**-**	**-**	**0**
**12**	**-**	**2.83**	**2.46**	**30.67**	**35.96**
**13**	**261.44**	**1.15**	**2.97**	**68.51**	**334.07**
**14**	**-**	**-**	**-**	**-**	**0**
**15**	**-**	**4.28**	**-**	**18.94**	**23.22**
**16**	**-**	**5.12**	**3.12**	**25.27**	**33.51**
**17**	**-**	**1.05**	**-**	**15.27**	**16.32**
**18**	**180.85**	**30.99**	**36.41**	**29.71**	**277.96**
**19**	**7.25**	**1**	**26.89**	**46.87**	**82.01**
**20**	**-**	**8.62**	**-**	**56.25**	**64.87**
**21**	**-**	**-**	**-**	**-**	**0**
**22**	**-**	**5.64**	**-**	**34.12**	**39.76**
**23**	**-**	**-**	**-**	**-**	**0**
**24**	**146.15**	**0.17**	**48.41**	**6.36**	**201.09**
**25**	**-**	**-**	**-**	**-**	**0**
**26**	**-**	**0.54**	**-**	**26.62**	**27.16**
**27**	**-**	**1.26**	**-**	**25.45**	**26.71**
**28**	**-**	**0.46**	**32.85**	**19.12**	**52.43**
**29**	**65.97**	**0.39**	**4.08**	**70.44**	**140.88**
**30**	**-**	**-**	**-**	**-**	**0**

**Fig. 1:**
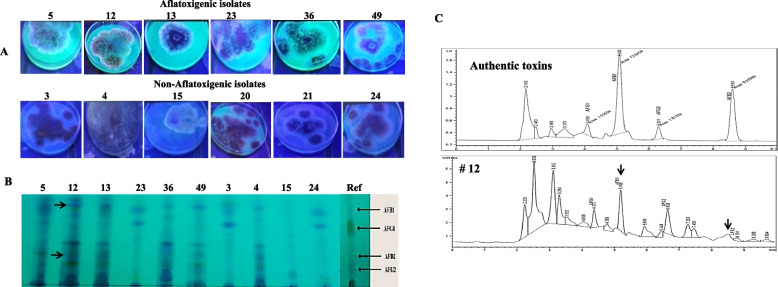
Screening for aflatoxins production by the selected isolates of *Aspergillus flavus*. A, Preliminary visualization of aflatoxigenic potency of *A. flavus* isolated from wheat grains by UV illumination at λ365 nm. B, TLC of the selected isolates of *A. flavus,* compared to authentic aflatoxins B1, B2, G1 and G2. C, HPLC chromatograms of the most potent aflatoxigenic isolates of A. flavus # 12, 23, and 36

The aflatoxigenic fungi inhabiting the thirty collected samples of wheat grains from the different local stores were isolated on PDA and Czapek’s-Dox media. Twenty-two species belonging to nine genera namely; *Alternaria*, *Aspergillus*, *Botryotrichum*, *Cladsporium*, *Fusarium*, *Penicillium*, *Rhizopus*, *Trichoderma* and *Ulocladium* were identified from the thirty wheat grains samples (Data not shown), among these isolates, only the fungal isolates belongs to *Aspergillus flavus* group were considered for further aflatoxin producing potency assay. From the results (Table S1), seventy fungal isolates belonging to *Aspergillus flavus* group were collected from the thirty wheat grains samples, these isolates mainly belong to three species *A. flavus,* A. *aflatoxiformans* and *A. parasiticus* species. The recovered fungal isolates were grown on Yeast extract-sucrose agar medium by centrally inoculated plug, incubated for 7 days, and then their aflatoxigenic producing potency was assessed by illumination to plate culture with UV at λ_365_ nm. From the results (Fig. 1), seven fungal isolates of *A. flavus* group displayed an obvious blue and green fluorescence emission around the fungal colony, and their surrounding media, suggesting the diffusion of the aflatoxins via the agar media away from the colony, unlike to the negative fluorescence emitted fungal colonies of *A. flavus* isolates. The emission of blue and green fluorescence could be a preliminary noticeable indicator for the aflatoxigenic producing potency of fungi. The positive fluorescence emitted fungal colonies isolates #, 10, 12, 13, 23, 38, and 49, while, the non-aflatoxigenic isolates # 3, 4, 9, 15, 20 and 21, had no fluorescence emission. For confirming the aflatoxigenic producing potency, the recovered isolates of *A. flavus* group (70 isolates) were grown on Yeast extract-sucrose broth medium for 10 days, then the cultured were filtered, and the aflatoxins were extracted and quantified by TLC and HPLC. From the obtained results (Table S1), out of 70 isolates belonging to *Aspergillus flavus* group obtained from the wheat grains samples, 50 isolates (71.4%) were found to be toxigenic isolates. The most active aflatoxins producing isolates were the isolate # 12, in which the yield of aflatoxins B_1_, and B_2_, was 517.8, and 22.5 μg/L, respectively, followed by the isolate # 23, producing a substantial amounts of aflatoxins B_1_ (404.2 μg/L) and B_2_ (10.4 μg/L). The yield of aflatoxins B_1_ and B_2_ by the isolate # 36 was 259.3 and 24.1 μg/L, respectively. Thus, the highest aflatoxins isolate were #12, followed by 23 and 36, as revealed from the TLC and HPLC profile. Interestingly, the highest aflatoxins producing isolates # 12 was recovered from the highest aflatoxin containing wheat grains. The HPLC chromatograms of the three potent aflatoxigenic isolates (#12, 23 and 26), illustrates the maxima of aflatoxins B_1_ and B_2,_ regarding to the retention time and concentration of the authentic aflatoxins (Fig. 1). The productivities of aflatoxins among the isolates of *A. flavus* group was highly fluctuated from zero to significant toxins producers, not only among the isolates collected from the different wheat grains samples, but also among the isolates from the same wheat grains sample. Thus, the biosynthetic machinery of aflatoxins among the recovered isolates of *A. flavus* was greatly dependent on various environmental conditions and native surrounding microbiome of the sample. Thus, the highest toxin producing isolate #12 from the highest toxin containing wheat grains #7, could be due to the improper storage conditions allowing to the growth of this aflatoxigenic isolate. Thus, the most potent aflatoxins producing isolates # 12, 23 and 26 were morphologically identified and molecularly verified.

### Morphological and molecular identification of the most active aflatoxin producing fungal isolates

**Fig. 2:**
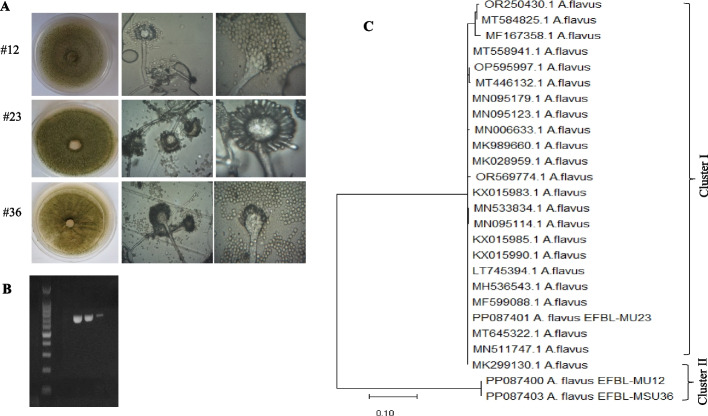
Morphological and molecular identification of the most active aflatoxins producing isolates of *A. flavus*. **A** Plate culture and conidial heads of isolate # 12, 23 and 36. **B** PCR amplicon of the ITS regions of the three aflatoxins producing isolates. **C** Molecular phylogenetic analysis of isolate # 12, 23 and 36, by Maximum Likelihood method. The microscopic view of the conidial heads of the three isolates by the light microscope at magnification 400 X and 1000X

The morphological features of isolate #12, 23 and 36 based on the conidial heads, conidial ontology, conidiophores identity, type of strigma, closely follows the morphological descriptions of *A. flavus*, according to the reference identification keys [34, 63]. The morphological features of the potent isolates are shown in Fig. 2. The morphological identity of the potent aflatoxins producing isolates were further confirmed based on their ITS sequence, using fungal genomic DNA as template for PCR. The PCR amplicon of the ITS regions were about 700-800 bp (Fig. 2). These amplicons were sequenced, and searched on non-redundantly BLAST searched on the NCBI database, gave 100 % similarity with the data-base deposited ITS sequences of *A. flavus* with 100% query coverage and zero E-value. The sequences of the ITS regions of the isolates *A. flavus* EFBL-MU12, EFBL-MU23 and EFBL-MU36 were deposited to the Genbank with accession # PP087400, PP087401, and PP087403, respectively. From the alignment profile and phylogenetic analysis of ITS sequences, the ITS sequences were grouped into two clusters I and II (Fig. 2). The ITS sequence of the *A. flavus* EFL-MU12 PP087400, and EFBL-MU36 PP087403 belongs to the same cluster II, with about 99.5 similarities with *A. flavus* with deposition # MK299130.1, MN511747, MF599088.1, LT745394.1, KX015990.1, KX015985.1, MN095114.1, MN533834, KX015983.1, OR569774.1, MMK028959.1, MN006633.1 and MN095179.1, with E value zero and 99 % query coverage. Thus, from the morphological description and molecular fingerprint, the current isolates were identified as *A. flavus.* The slight morphological changes in the shape of the conidial heads, macromorphological features, and conidial fructification on the plate cultures, might be related to intrinsic isolate-isolate physiological variations.

### Bioprocess optimization of Aflatoxins production by *A. flavus* by Plackett-Burman design grown on Wheat grains under solid date fermentation

**Fig. 3:**
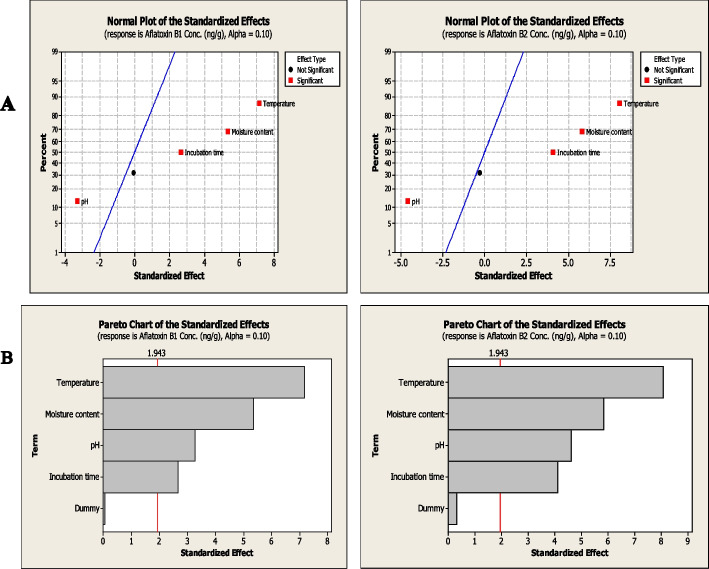
Nutritional optimization of aflatoxins (B1 and B2) production by *Aspergillus flavus #*12 with the Plackett-Burman experimental and FCCD designs. A: Normal probability plots of the standardized effects of the variables on production of aflatoxins B_1_ and B_2._ B: Pareto charts illustrating the significance of each variable on production of aflatoxins B_1_ and B_2_

Since the storage conditions is a critical factors affecting the growth of aflatoxigenic fungi and subsequent aflatoxin synthesis, so the nutritional optimization of the medium components to assess their influence on aflatoxins production *A. flavus* has been assessed. The growth of *A. flavus* EFBL-MU12 and their aflatoxins productivity was optimized by Plackett-Burman design as 1st order model equation. Four parameters of the physical factors; temperature, moisture content, pH and incubation time were studied on aflatoxins production by the fungal isolate, with their lower and higher values depending on the preliminary experimental results affecting the growth of this isolate (Table 2). The significant independent parameters affecting aflatoxins production by *A. flavus* with the predicted and corresponding actual responses were summarized in Table S1. From the obtained data, the highest yield of aflatoxins B_1_ and B_2_ were 145.3, 7.6, 16.4 and 25.6 μg/kg, respectively, has been reported at run # 6, followed by run # 7 and 11. While an obvious fluctuations and variations on the aflatoxins productivity by the tested fungal isolate, has been observed for the different Plackett-Burman designed runs. The experimental responses were analyzed ANOVA using Minitab 16 software, to detect the most significant factors affecting aflatoxin production at 90 % level of confidence and α 0.1 (Table S2). From the Placket-Burman design, the tested factors had a statistically significant effect on the production of aflatoxins B_1_, and B_2,_ as reveled from the values of Fisher’s F-test 19.5 and 17.4, with the *p*-value 0.0001 and 0.002. The normal probability plots and Pareto charts of the standardized effects of the variables on production of aflatoxins B_1_ and B_2_ were shown in Fig. 3. Obviously, the pH had a statically significant negative effect on production of aflatoxin B_1_ and B_2_, while, the other tested factors showed a positive significant effect with *p*-value <0.1. The effect of variable on each computed response with the factors crossing the red line to be factually significant was illustrated from the Pareto Chart (Fig. 3). So, it was clearly from the Plackett-Burman design, the highest productivity of aflatoxins by *A. flavus* grown on wheat grains was reported at temperature 35°C, 16% moisture contents, initial pH 5.0, and incubated for 14 days.

**Table 2:** Matrix and responses of the CCD for the significant physical factors affecting aflatoxins production by the isolate # 12

**Runs**	**Temperature**	**Moisture content**	**pH**	**Incubation time**	**Aflatoxin B**_**1**_ **(μg/kg)**	**Aflatoxin B**_**2**_ **(μg/kg)**
1	27.5	15	6.5	26	117.32	18.22
2	20.0	10	5.0	20	102.56	7.35
3	27.5	15	3.5	14	79.19	8.78
4	20.0	20	5.0	20	137.92	13.43
5	27.5	15	6.5	14	178.01	20.12
6	35.0	20	5.0	8	80.08	2.65
7	27.5	5	6.5	14	40.00	0.99
8	20.0	20	8.0	20	109.58	7.99
9	35.0	10	8.0	20	52.08	3.00
10	27.5	15	6.5	14	183.91	21.78
11	27.5	15	6.5	14	183.99	23.22
12	27.5	15	6.5	14	200.16	25.43
13	27.5	15	6.5	14	173.01	21.88
14	12.5	15	6.5	14	47.33	4.92
15	27.5	15	6.5	2	10.11	0.36
16	27.5	15	6.5	14	169.59	23.11
17	35.0	10	5.0	8	65.06	7.06
18	20.0	10	8.0	20	33.17	1.33
19	20.0	10	5.0	8	20.69	0.98
20	27.5	15	6.5	14	173.94	22.09
21	27.5	15	9.5	14	45.98	2.35
22	35.0	20	5.0	20	144.21	12.22
23	20.0	10	8.0	8	12.71	2.00
24	35.0	20	8.0	8	99.13	9.06
25	20.0	20	8.0	8	62.00	5.76
26	35.0	20	8.0	20	120.51	11.33
27	35.0	10	8.0	8	60.89	5.19
28	35.0	10	5.0	20	139.12	16.14
29	27.5	25	6.5	14	155.61	8.24
30	42.5	15	6.5	14	110.27	17.99
31	20.0	20	5.0	8	70.69	1.74

### Optimizing the aflatoxins productivity of *A. flavus* EFBL-MU12 with Central Composite Design

**Fig. 4:**
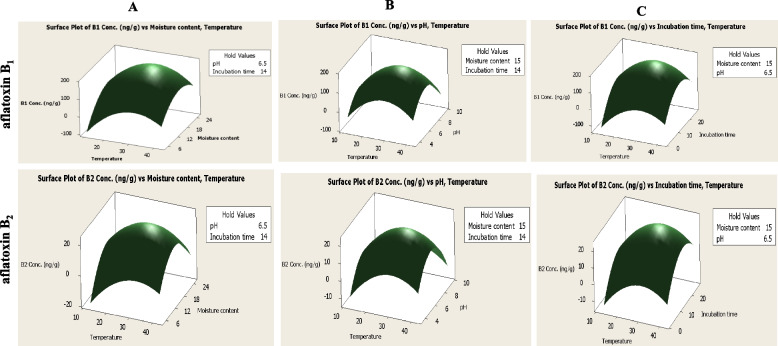
The three-dimensional surface plots for interaction between temperature and moisture content (**A**), temperature and pH (**B**), temperature and incubation time (**C**), for the optimal yield of aflatoxin B_1_ and B_2_ by *A. flavus* from the Plackett-Burman and FCCD

The Central Composite Design (CCD) was assesses the interactions of variables “temperature, moisture content, pH and incubation time” on aflatoxins production by *A. flavus* grown on wheat grains. Each variable has five levels as shown in Table 2. The maximum productivity of aflatoxins B_1_ and B_2_ with 200.1, 25.4 μg/kg, respectively, was reported at the trial # 12, in which the incubation temperature 27.5°C, moisture content 15%, pH 6.5, and incubated for 14 days. Analysis of main effect, quadratic effects and interaction between factors were summarized (Table S2). The factors with *p*-value < 0.1 were considered to be significant. From the CCD analysis, the model of aflatoxins B1 and B2 was considered to significant as reveled from the values of F-test 53.0, and 26.2, respectively. The most significant factors affecting aflatoxins production was reported at 90 % level of confidence, with r^2^ value 97.8, and 95.8 % for the aflatoxins B_1_ and B_2_, revealing the goodness of fit of the regression model. The main effect of the four factors in addition to quadratic effect of them were found to be significant factors affecting aflatoxins production with *p*-value <0.1. The 3D response surface plots illustrating the defined the effect of interaction of the factors on the production of aflatoxins B_1_ and B_2,_ were shown in Fig. 4. The interaction between temperatures, moisture contents, pH and incubation times were found to be a significant factors affecting aflatoxins B_1_ and B_2_ production, while the interaction of moisture content and pH, as well as pH and incubation time were the most significant for affecting aflatoxin B_2_ production. Thus, from the results of evaluating the effects of physical factors of storage of wheat grains with *A. flavus,* by the response surface methodology, it is clearly showed that the storage humidity, temperature and incubation periods of the wheat grains have a significant impact on the aflatoxins productivity and identity by *A. flavus.*

### Effect of plant essential oils on growth and aflatoxins production by *A. flavus*

**Fig. 5.**
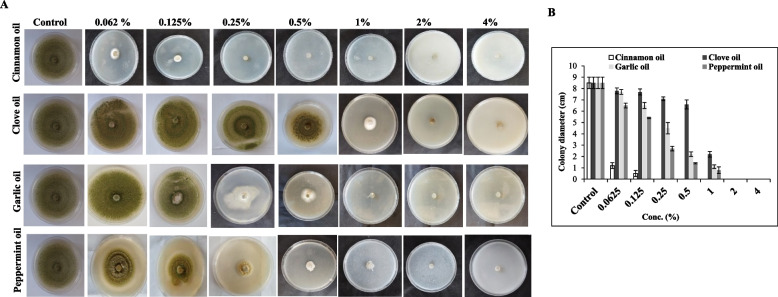
**A** Plate culture of *A. flavus* in response to different concentrations (0, 0.062, 0.125, 0.25, 0.5, 1.0, 2.0 and 4.0%) of cinnamon oil, clove oil, garlic oil and peppermint oil. **B** Linear growth of *A. flavus* in response to different concentrations of the tested essential oils, as expressed by the colony diameters (cm)

The effect of essential oils of cinnamon oil (*Cinnamomum zeylanicum*), clove oil (*Syzygium aromaticum*), garlic (*Allium sativum*), Pepper mint (*Mentha peperita*) on the growth and aflatoxins production of *A. flavus* EFBL-MU12 was assessed. As shown from the radial growth of the plate cultures of *A. flavus* (Fig. 5), the tested essential oils have a fungicidal effect on growth of *A. flavus* in a concentration-dependent manner. Among these essential oils, cinnamon oil had the highest fungicidal effect on growth of *A. flavus* at 0.25%, while at 0.062 and 0.125%, the fungal growth was inhibited by about 86.3% and 94.4%, respectively (Table 3). While, the other essential oils; clove, garlic and peppermint oils displayed a relatively similar inhibitory pattern as revealed from the radial fungal growth on the plate cultures. The fungal growth was reduced by about 75.2, 87.4, and 90.7 %, in response to clove, garlic and peppermint oils, respectively, at oil concentrations 1%, with a clear absence of fungal growth at 2 and 4% (Table S3). The minimum inhibitory concentration (MIC) of the experimented oils were calculated from the fungal radial growth, showing that the MIC of cinnamon oil was 0.2 %, while for clove, garlic and peppermint oils, it was 2%. So, from the MIC values, the cinnamon oils had an antifungal activity towards *A. flavus* by about ten folds higher than the other tested oils.

**Table 3:** Effect of different concentrations of the tested essential oils on the aflatoxins production by isolate # No.12 in wheat grains

**Conc. of essential oil (%)**	**Cinnamon oil**		**Clove oil**		**Garlic oil**		**Pepper mint oil**	
	**B**_**1**_** (μg/kg)**	**B**_**2**_** (μg/kg)**	**B**_**1**_** (μg/kg)**	**B**_**2**_** (μg/kg)**	**B**_**1**_** (μg/kg)**	**B**_**2**_** (μg/kg)**	**B**_**1**_** (μg/kg)**	**B**_**2**_** (μg/kg)**
**Control**	**356.6**^**a**^**±6.7**	**133.4**^**a**^**±5.8**	**356.6**^**a**^**±6.7**	**133.4**^**a**^**±5.8**	**356.6**^**a**^**±6.7**	**133.4**^**a**^**±5.8**	**356.6**^**a**^**±6.7**	**133.4**^**a**^**±5.8**
**0.0625%**	**257.4**^**b**^**±7.2**	**97**^**b**^**±5.8**	**261.03**^**b**^**±5.4**	**117**^**b**^**±4.4**	**204.1**^**b**^**±5.5**	**126.7**^**ab**^**±2.4**	**309.8**^**b**^**±6.1**	**132.5**^**a**^**±4.5**
**0.125%**	**93.5**^**c**^**±6**	**60.9**^**c**^**±5**	**153.3**^**c**^**±4.7**	**82.5**^**c**^**±5**	**149.7**^**c**^**±4.5**	**120.5**^**b**^**±2.3**	**287.4**^**c**^**±4.5**	**116.6**^**b**^**±1.3**
**0.25%**	**-**	**-**	**61.2**^**d**^**±5.2**	**52.7**^**d**^**±4.8**	**130.1**^**d**^**±4.4**	**101.1**^**c**^**±4.3**	**251.3**^**d**^**±3.6**	**95.1**^**c**^**±5.2**
**0.5%**	**-**	**-**	**57.3**^**de**^**±6.4**	**31.4**^**e**^**±3.9**	**115.8**^**e**^**±5.1**	**59.2**^**d**^**±4.3**	**165.1**^**e**^**±4.9**	**62.2**^**d**^**±2.1**
**1%**	**-**	**-**	**50.8**^**e**^**±4.3**	**17.3**^**f**^**±2.2**	**84.9**^**f**^**±4**	**33.2**^**e**^**±5.6**	**107.4**^**f**^**±2.4**	**33**^**e**^**±2.4**
**2%**	**-**	**-**	**18.4**^**f**^**±3.6**	**6.9**^**g**^**±0.9**	**52.03**^**g**^**±7.03**	**10.4**^**f**^**±2.8**	**65.2**^**g**^**±5.1**	**15.4**^**f**^**±2.2**
**4%**	**-**	**-**	**-**	**-**	**-**	**-**	**-**	**-**
**LSD**	**13.23**	**11.11**	**9.27**	**7.28**	**9.49**	**7.29**	**8.64**	**6.49**

Each value represents by the mean of 3 replicates (Mean ± SD). The same letters in each column represents insignificant difference where LSD at *p* ≤ 0.05

The antifungal activity of the tested essential oils towards *A. flavus,* was evaluated from the conidial pigmentation, in response to different concentrations of the oils. After incubation of the culture media of *A. flavus* containing different oils concentrations, the melanin pigment of the fungus was extracted and quantified. From the results (Fig. S1), cinnamon oil exhibited the significant effect on inhibiting the conidial pigmentation of *A. flavus*, the conidial heads color changed from yellow-green to white at 0.0625%, with significant suppression to the overall growth and melanin amounts at 0.2% oils (Table S4). The overall reduction in melanin concentration follows the reduction in the fungal biomass in a concentration dependent manner of oil. For the clove and peppermint oil, the maximum decrease in melanin concentration was recorded at 1% yielding 10% and 5% for clove and peppermint respectively.

### Scanning electron microscope analysis of *A. flavus* in response to the essential oils

**Fig. 6:**
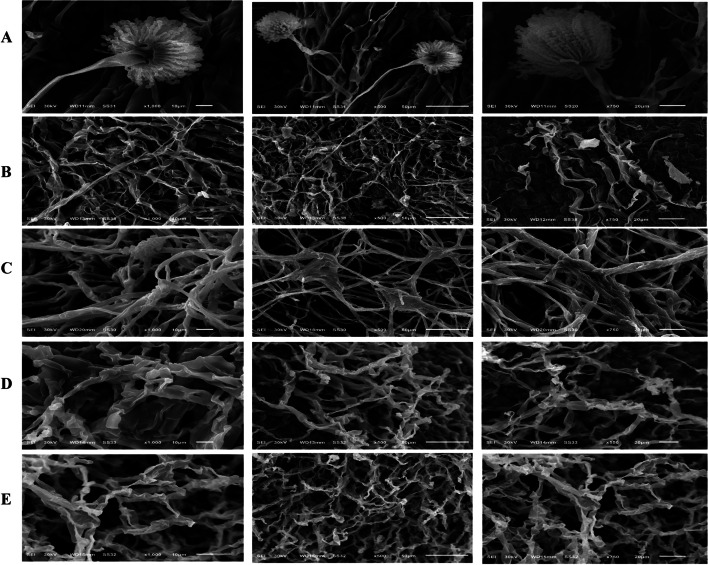
SEM of *A. flavus* in response to different concentrations of the tested essential oils. **A** Control of *A. flavus* (Zero oil), **B**: Treatment with 0.12% cinnamon oil, **C**: Treatment with 1% clove oil, **D**: Treatment with 0.5% garlic oil, and **E**: Treatment with 1 % peppermint oil

The morphological aberrations of the aflatoxigenic isolate *A. flavus* in response to the sub-MIC values of the tested oils were assessed, by amendment the plate culture with desired concentration. The sub-MIC values of the tested oils of cinnamon (0.12%), clove (1%), garlic (0.5%) and peppermint (1%), were amended to the solid medium prior pouring in plates. From the SEM analysis (Fig. 6), an obvious morphological aberration to the conidial heads, and vegetative mycelia, alterations in the shapes of conidiophores and substrate mycelia, were appeared especially in response to cinnamon oils, followed by other oils. For the control sample, the mycelia showed a normal morphology and the hyphae were linear, regular and homogenous, and their cell walls were smooth, with distinct oval conidial heads, bi-seriate strigma, and visual oriented conidial chain. The vegetative mycelia and conidiophores anastomosis underwent alterations upon exposure to the tested oils, the mycelial tips were elongated underwent morphological abnormalities, hyphae shrank, underwent winding, losing their linearity.

### Effect of the essential oils on the growth and aflatoxins production by *A. flavus*

**Fig. 7:**
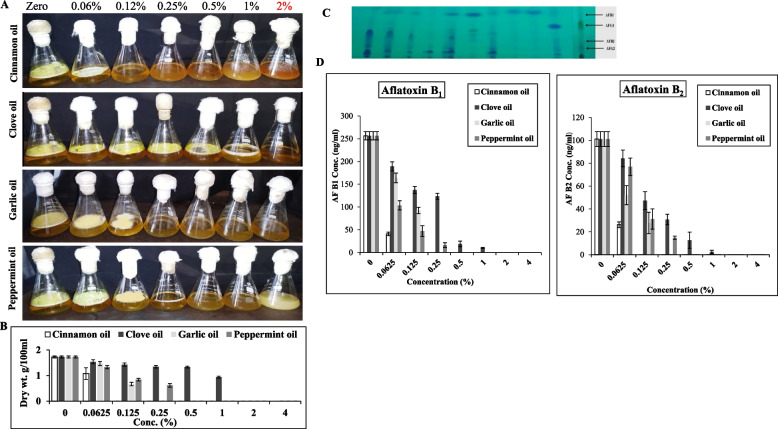
**A** Cultures of *A. flavus* on yeast extract sucrose broth media in presence of essential oils of cinnamon, clove, garlic and peppermint at different concentrations. **B** The fungal biomass in response to the different concentrations of the tested essential oils. The fungal cultures were incubated at standard conditions, then the aflatoxins were extracted and fractionated by TLC, and their concentrations was determined. **C** TLC plate of the aflatoxins, compared to the authentic ones. **D** The yield of Aflatoxins B1, and B2, as estimated by the TLC and confirmed by HPLC

The potency of the tested essential oils on biosynthetic potency of aflatoxins by *A. flavus* has been evaluated. The liquid media was amended with different concentrations of the essential oils, after incubation, the mycelial biomass and aflatoxins producing potency was assessed by the TLC and HPLC. From the results (Fig. 7), the visual growth of *A. flavus* was reduced with the higher essential oil concentrations. Interestingly, the fungal growth was completely inhibited with 0.12% cinnamon oil, however, the other oils exhibited about 50% reduction on the mycelial biomass at this concentrations (0.12%), ensuring the superior efficiency of cinnamon oils than the oils. Following cinnamon oil, garlic oil showed a significant inhibitory effect on fungal biomass by about 14.7 and 61.1%, at oil concentration 0.062 and 0.125%, respectively, compared to control, while at 0.25 and to 4%, the fungal growth was completely inhibited. Clove oil was the least effective oil on the growth of *A. flavus* showing a significant reduction at 1 %, with 45.1 % inhibition.

The effect of essential oils on aflatoxins production by *A. flavus* EFBL-MU12 was estimated, after growing of the isolate on yeast extract broth media, the aflatoxins were extracted and quantified by TLC and HPLC. From the results (Fig. 7), the productivity of aflatoxins were strongly suppressed with the essential oils concentrations, with a drastic inhibition to the growth and subsequent aflatoxins production with cinnamon oil, followed by garlic oils, peppermint oils and clove oils. The production of aflatoxins B_1_, and B_2_ by *A. flavus* was significantly decreased at 0.125 % of garlic oil by about 64 and 72.5%, respectively, compared to the control. The peppermint oil showed the maximum reduction of aflatoxins B_1_ and B_2_ production by *A. flavus* at 0.25% by about 93.6 and 85.3%, respectively. Upon using of clove oils, the maximum decrease in aflatoxins productivity was reported at 1%, by about 96.1 and 97.6 %, for aflatoxins B_1_ and B_2_, respectively. Conclusively, cinnamon oil had the most fungicidal effects on growth and aflatoxins production by *A. flavus* at 0.0625, compared to the control (Fig. 7).

### Effect of plant essential oils on growth and aflatoxins production by *A. flavus* in wheat grains

**Fig. 8:**
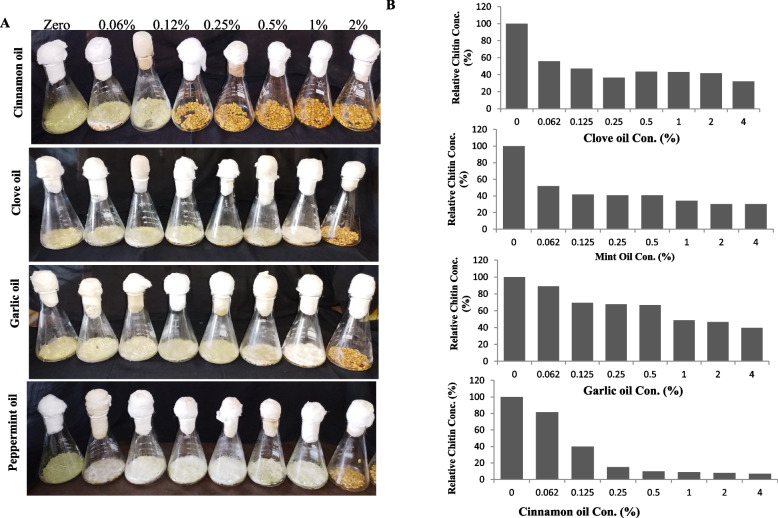
Growth of *A. flavus* on wheat grains. **A** Visual growth of *A. flavus* on wheat grains under solid state fermentation in presence of different oils concentrations. **B** Chitin concentrations of the entire cultures of *A. flavus* grown on wheat grains

The impact of essential oils on growth and aflatoxins production by *A. flavus* grown on wheat grains under solid state fermentation was assessed. After incubation of the fungal cultures at the desired conditions, the fungal growth was determined in terms of total chitin contents, as well as, the aflatoxins productivity was assessed by the TLC and HPLC. From the visual growth (Fig. 8), a noticeable suppression in the apparent growth of *A. flavus* on wheat grains under solid state fermentation with the oils concentration in a concentration-dependent manner. The highest antifungal activity was reported for cinnamon oils, compared to other essential oils. The growth of *A. flavus* was completely disappeared at 0.12 % of cinnamon oil, however, an obvious fading on the fungal pigmentation, with whitish mycelium without, absence of conidial heads and lack of fungal sporogenesis at concentrations till 1 % oils concentrations.

As well as, the fungal growth was assessed from the total chitin contents of the solid state cultures on wheat grains. The chitin contents of *A. flavus* grown on wheat grains was reduced by about 75 % and 95 % at 0.125 and 0.25%, respectively, of cinnamon oil, compared to control (zero essential oil). However, a relatively similar reduction pattern of chitin contents by about 50% at 1% of clove, garlic, and mint essential oils, suggests the relative growing of the fungus but without visual melanin pigmentation and spores’ formation. Interestingly, the reduction in the visual growth, and fading of the mycelial pigmentation were clearly matched with the reduction on chitin contents of the fungus grown on wheat grains as solid state fermented medium.

**Fig. 9:**
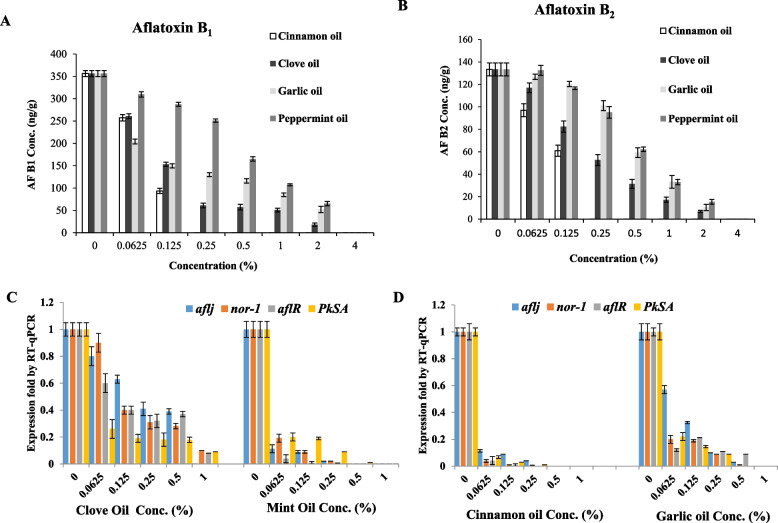
Aflatoxins production and molecular expression analysis of the aflatoxins biosynthetic genes of *A. flavus* grown on wheat grains in response to different oils concentration. **A**, **B** The yield of aflatoxins B1, and B2 by *A. flavus* on wheat grains solid fermented cultures in response to different concentrations of the essential oils. B, Molecular expression analysis of the aflatoxins biosynthetic genes *aflJ*, *nor-1*, *aflR* and *pksA* in response to clove and peppermint oils (**C**), and in response to cinnamon and garlic oils (**D**), normalizing to *actA* gene as a house keeping gene

The productivity of aflatoxins by *A. flavus* grown on wheat grains was evaluated, after incubation of the cultures, aflatoxins were extracted and quantified by the TLC and HPLC (Fig. 9). Practically, the productivity of aflatoxin B_1_, and B_2_ by *A. flavus* grown on wheat grains was significantly decreased with the higher concentration of the tested essential oils. Cinnamon oil displayed the highest inhibitory effect on aflatoxins biosynthesis, at 0.125% of oil, the yield of aflatoxin B_1_ and B_2_ was approximated by 93.5±6, 60.9±5 μg/kg, i.e reduced by about 73.8%, and 54.3%, respectively, compared to control. However, for clove, garlic and peppermint oils, the maximum suppression of aflatoxins biosynthesis by *A. flavus* EFBL-MU12 was recorded at 2% for these oils. The yield of aflatoxin B_1_ upon using clove oil at 2% was significantly reduced to 18.4 μg/kg, by about 94.8%, followed by garlic and peppermint oils, in which the aflatoxins yield were reduced by 85.4% and 81.7%, respectively, compared to control. The yield of aflatoxin B_2_ biosynthesis in response to clove oil, garlic oil and peppermint oil was reduced by 94.8, 92.2 and 88.5 %, respectively, at 2 % of oils..

### Molecular expression analysis of the aflatoxin biosynthetic and regulatory genes expression of *A. flavus*, in response to essential oils treatment

The molecular expression of the rate limiting genes and regulatory genes of aflatoxin biosynthesis of *A. flavus* grown on yeast extract sucrose broth media under submerged state fermentation was estimated. The fungal isolate was grown on yeast extract sucrose broth media amended with the tested oils of cinnamon, clove, garlic and peppermint at different concentrations, then incubated for 10 days, at 28°C, in addition to the control cultures (without oil). The total RNA was extracted from the cultures, revers transcribed into cDNA, and the molecular expression of the rate-limiting genes and regulatory genes was assessed by qPCR normalized to *actin A,* as house-keeping gene. Blank media without fungal inocula were used as a baseline for qPCR calculations. The molecular expression folds of the *afLJ*, *nor-1*, *afLR* and *pKsA* genes were shown in Fig. 9. From the relative expression folds, the expression of the aflatoxins synthetic and regulatory genes were significantly suppressed in response to the tested oils, compared to the control cultures. The expression of the *nor-1, afLR*, *pKsA* and *afLJ* genes of *A. flavus* were significantly down regulated by about 94-96%, in response to treatment with cinnamon oil at 0.062 %, compared to control cultures. Also, the expression of the *nor-1, afLR*, *pKsA* and *afLJ* genes of *A. flavus* upon using peppermint oils were reduced by about 92-94%, compared to control cultures. However, the clove and garlic oils had a relatively similar pattern of down regulation of the *nor-1, afLR*, *pKsA* and *afLJ* genes of *A. flavus,* by about 60-85%, normalized to control. So, the overall reduction of the fungal visual growth, chitin contents, and aflatoxins yields by TLC and HPL, was matched with the down regulation of the molecular expression levels of aflatoxins biosynthetic genes, in response to treatment with essential oils. Conclusively, from the results, cinnamon oils followed by the peppermint oils displayed the most fungicidal activity for the growth and aflatoxins production by the aflatoxigenic isolate *A. flavus* under solid-state fermentation conditions on wheat grains.

### Metabolic profiling of the solid state-fermented cultures of *A. flavus* grown on wheat grains in response to the tested essential oils

The metabolic profiling of the solid-state fermented cultures of *A. flavus* grown on wheat grains in response to the selected essential oils were estimated, compare to the control cultures (without oils). The cultures were amended with different concentrations of the cinnamon oils and clove, then after incubation at the desired conditions, the cultures were extracted by methanol, and the metabolic profiling was determined by GC-MS analysis. From the metabolic profiling (Table 4), some metabolites were sequentially reduced upon addition of cinnamon and clove oils, compared to the control cultures, such as Dehydromevalonic lactone, octadecanoic acid, 3-methoxy-2-(1-methylethyl)-5-(2-methylpropyl) pyrazine, pentanoic acid and cyclohexane-carboxaldehyde. These compounds were strongly reduced with addition of essential oils, with an obvious correlation with the aflatoxins productivity, suggesting their implications as intermediates on the biosynthetic pathways of various polyketides particularly aflatoxins. As well as, from the metabolic profiling, several metabolites were completely disappeared from the control cultures upon addition of the essential oils at the tested concentrations such as 3-Nitro-1-phenylheptan-1-ol, Deoxyspergualin, 5,11,17, 23-TETRAKIS (1,1-DIMETH YLETHYL)-28 METHOXYP ENTACYCLO and 18-Pentatriacontanone, suggesting the implementation of these metabolites on different metabolic pathways related to fungal growth and bioactive metabolites productivities.

**Table 4:** Metabolic profiling of the solid cultures of *A. flavus* grown on wheat grains by GC/MS

**Compound name**	**control**	**Cinnamon oil**		**clove oil**	
		**0.062 %**	**0.125 %**	**0.062 %**	**0.25 %**
Dehydromevalonic lactone	4.12	0.98	0.21	0.99	-
3-Nitro-1-phenylheptan-1-ol	0.12	-	-	-	-
3,4-DIHYDRO-2H-1,5-(3-T-BUTYL) BENZODIOXEPINE	0.97	0.9	0.48	0.62	0.34
3,4-Dihydrocoumarin, 6-fluoro-4,4-dimethyl	0.58	0.34	0.13	-	-
6,12-EPOXY-11á-EUDESMA -4,6-DIEN-3-ONE	0.28	0.22	0.21	-	0.23
3-Methoxy-2-(1-methylethyl)-5-(2-methylpropyl) pyrazine	1.11	0.29	0.15	0.93	0.26
Pentanoic acid	1.11	0.14	0.09	0.26	0.18
4-(2-ACETYL-5,5-DIMETHY-CYCLOPENTEN-1-YLID ENE)-2-BUTANONE	1.61	-	0.48	-	0.50
3-(1-HYDROXY-2-ISOPROP-METHYLCYCLO-HEX YL)-2-PROPYNOIC ACID	0.59	0.13	0.06	-	-
Erucic acid	0.17	-	0.16	-	0.15
12-HYDROXY-14-METHOXY -3-METHYL-3,4,5,6,7,8,9,10-O CTAHYDRO-1H-2-BENZOXA	0.9	0.13	0.11	-	-
4H-1-BENZOPYRAN-4-ONE	0.16	0.15	0.09	-	0.14
Propiolic acid	0.47	0.25	0.12	0.31	0.11
Cyclohexanecarboxaldehyde	10.29	3.85	0.17	-	1.27
13-Heptadecyn-1-ol	0.27	0.16	0.11	-	-
Deoxyspergualin	0.17	-	-	-	-
7,9-Di-tertbutyl-1-oxaspiro-deca-6,9-diene-2,8-dione	0.81	0.49	0.42	-	0.43
1,2-Benzenedicarboxylic acid, butyl octyl ester	0.85	0.69	0.21	-	0.14
1,3,6,10-Cyclotetradecatetraene	0.46	0.41	0.35	-	0.37
n-Hexadecanoic acid	6.64	1.66	1.24	1.78	0.37
HEXADECANOIC ACID	0.51	0.19	0.13	-	0.12
Hexadecanoic acid, ethyl ester	0.76	0.49	0.68	-	0.75
9-OCTADECENOIC ACID	0.26	0.22	0.37	-	0.24
Oleic Acid	0.34	0.27	0.22	-	0.1
OCTADECANOIC ACID, METHYL ESTER	18.32	0.94	0.60	0.72	0.63
3,4-DIMETHOXYPHENETHY	0.54	0.29	-	0.51	0.16
cis-13-Octadecenoic acid	5.03	3.31	3.27	0.27	0.16
Octadecanoic acid	11.25	7.51	6.90	2.64	0.19
OCTADECANOIC ACID, ETHYL ESTER	3.38	3.14	1.82	3.11	2.33
OCTADECANOIC ACID	5.29	0.16	0.12	0.34	-
Glycidyl palmitate	4.72	4.23	3.15	3.06	2.98
9-OCTADECENAMIDE	0.77	0.48	0.38	0.69	0.45
Octadecanamide	3.88	3.86	2.87	2.51	0.60
Octadecanoic acid	0.34	0.3	0.26	0.21	-
9-Octadecenoic acid (Z)-, oxiranylmethyl ester	2.12	0.79	0.78	1.03	0.77
Glycidyl palmitate	17.84	11.43	10.57	13.56	13.33
10-METHOXY-NB-à- YLCORYNANTHEOL	0.63	0.33	0.23	0.16	-
9,12,15-OCTADECATRIENO IC ACID	1.51	0.75	0.32	-	0.26
9-OCTADECENOIC ACID (Z)	0.48	0.32	0.07	-	-
5,11,17 23-TETRAKIS(1,1-DIMETH YLETHYL)	2.41	-	-	0.16	-
3,7,11,15,19-Pentaoxa-2,20-disilaheneicosane	4.68	3.12	2.13	2.65	-
Uleine, N-demethyl-N-ethyl	1.52	1.60	0.38	-	-
9,12-OCTADECADIENOIC ACID (Z,Z)	1.19	0.2	0.12	-	0.12
3-(TRIMETHYLSILYLOXY)- 5,7-DIMETHOXY-	4.66	4.04	3.44	-	-
18-Pentatriacontanone	16.06	-	-	-	1.82

## Discussion

The increasing of the world population, with the obvious reduction in the accessibility of foods, food safety, food quality, and food security especially with the climatic and environmental changes add an extra load to the biotechnologist to search for a novel sources and approaches to combat these challenges for improving the global healthy life [64]. Cereals, including wheat are one of the major indispensable foods required by the world’s population, for both human and animal consumption. Storage conditions and the possibility of growth of aflatoxigenic fungi is of the most deleterious challenges that affecting not only the human and animal health but also on the global economy [65], by the production of mycotoxins. The possibility of growing the spoilage molds are a direct result to an open storage conditions, especially at the country of origin [66], that affected are by the environment, inappropriate handling, inconvenient drying and storage of grains during cultivation and harvesting. Thus assessment of aflatoxigenic fungi on the stored wheat grains, and possibility of stopping the growth of these fungi, and preventing their subsequent mycotoxins hazardous impacts was the objective of this study.

Seventy fungal isolates belonging to *Aspergillus flavus* group were isolated from the wheat grains, screened for aflatoxins production. Among these aflatoxigenic isolates, *A. flavus* EFBL-MU12, EFBL-MU23, and EFBL-MU36, were the most potent aflatoxins producers of B_1_, B_2_, G_1_ and G_2_. The morphologically identified isolates of *A. flavus* EFBL-MU12, EFBL-MU23, and EFBL-MU36 were molecularly confirmed based on the sequence of their ITS regions, the sequences were deposited on Genbank with accession # PP087400, PP087401, and PP087403, respectively. So, among the aflatoxins producing potency was visually varied among the different isolates of *A. flavus* recovered from the wheat grains which could be due to isolate-isolate variations, microbiome communication, and microbial interactions [67]. As well as, these variations on aflatoxins productivity could be due to the differences in expression of aflatoxin biosynthetic and regulatory genes between toxigenic isolates from cereals, that might be related to food storage and environmental conditions [68, 69]. The highest content of aflatoxins was detected in the wheat grains samples # 7, 13 and 18, which might be attributed to storage conditions of the samples, that being in consistent with those reported for wheat grains in Egypt, and found that AFB1 was detected in high concentrations in Al-Omraniyah and Atfih districts tin Giza governorate recording 35.21 and 49.79 μg/kg, respectively with slight higher humidity [70]. In addition, in the same study; aflatoxin B_2_ was detected in 75 % of the wheat grain samples, while AFG_1_ and AFG_2_ were detected in all of the wheat grain samples. Similar results reported that wheat grains having 16 % of the samples contaminated by AFB1 at 30 μg/kg that exceeded the European Commission [71]. Consistently, numerous studies reporting the presence of aflatoxins in wheat grains, ensuring the contamination with aflatoxigenic fungi [69, 72, 73] mainly due to the improper storage conditions of temperature, relative humidity, storage conditions, fungal growth, and insect pests.

The effect of storage conditions including temperature, moisture content, pH and incubation time on aflatoxins B_1_, and B_2_ production in wheat grains after inoculation with the selected isolate was studied by Plackett-Burman design and the significant variables were further optimized by the Central Composite Design (CCD). From the results, the tested factors have statistically significant effect on the production of aflatoxins by *A. flavus* EFBL-MU12, ensuring the highly significance of the model. The highest productivity of aflatoxins by *A. flavus* grown on wheat grains were reported at temperature 35°C, 16% moisture content, pH 5 and 14 days’ incubation. *Aspergillus flavus* poses an excellent survival capability to grow on a wide range of temperatures ranging from 12 to 48 °C, but the maximum growth and aflatoxins production were reported at 28 to 37°C [74]. Similar results documenting the maximum aflatoxins productivity by *A. flavus* were reported at the storage temperature of wheat grains of about 25-35°C [75]. Similarly, accumulation of high amounts of AFB1 was noticed at 20, 30 and 35°C with moisture content 25% in wheat flour [76]. Depending on the moisture contents necessities, the highest aflatoxins productivity of usually reported at wheat grain moisture of 13-18% (equivalent to 70% and 90% relative humidity) [77]. It had been reported that *A. parasiticus* was able to produce aflatoxins at 14% moisture content in wheat grains after 3 months of storage [78]. Aflatoxin-producing fungi can grow in a wide range of pH (1.7–9.3), but the optimum range of pH is (3–7) [79]. The lower pH 2.0-3.0, minimizes the fungal growth and a slightly higher pH 6.0-8.0 promotes the fungal growth and aflatoxins production [80]. Further studies with the CCD analysis, revealed that the aflatoxins B_1_, B_2_, G_1_ and G_2_ productivity were strongly significantly increased upon interaction of temperature and moisture content, that was coincident with those reported previously for *A. parasiticus* [52] grown on wheat flour. Therefore, grains with higher water content should be dried before storage to maintain seed viability, microbial stability, seed coat color and nutritional value. *Aspergillus flavus* usually contaminate food products and synthesizes aflatoxins as metabolites in the presence of elevated levels of carbohydrates and low levels of protein [81]. Aflatoxins production by *Aspergillus* section *Flavi* is firmly associated with high-carbohydrate and high-fat foods, for their possessing to a large diversity of hydrolytic enzymes [82].

Due to the harmful effects of aflatoxins, most research effort have concentrated on the means for prevention of AFs formation, by decontaminating methods for reducing the aflatoxin uptake through food chain [83]. Implementing of essential oils for controlling the growth of aflatoxigenic fungi and their subsequent aflatoxins production in cereal grains has received much attention and recommended. From the results, all the tested essential oils had an obvious effect on suppressing the growth of the aflatoxigenic *A. flavus* EFBL-MU12 grown on PDA, in a concentration dependent manner, compared to the control. Among the tested essential oils, cinnamon oil was the most active one inhibiting the growth of *A. flavus* at 0.0625-0.125%, with a subsequent stopping to the aflatoxins productivity. Similar results ensure the fungicidal effects of cinnamon, clove, peppermint, and garlic oils against different aflatoxigenic microbial species [84, 85]. The antifungal activity of *Syzygium aromaticum* and *Cinnamomum zeylanicum*, essential oils against *A. niger*, *A. ocharceus*, and *A. oryzae* from wheat bread were reported and *C. zeylanicum* EO showed maximum antifungal activity against all the fungal species having the largest inhibition zone at 800 mg/ml [86]. The tested oils significantly reduced melanin concentration in which cinnamon oil was the most active one reducing melanin concentration to 6 % at 0.125%. The fungal melanin, representing high-molecular hydrophobic pigments is synthesized via the polyketide synthase pathway of aflatoxins production [87]. Blocking the melanin biosynthesis in fungi resulted in the loss of their pathogenicity and increased fungal susceptibility to biotic and abiotic stresses [88–91]. The fluctuation of the inhibitory responses to the essential oils to toxigenic *A. flavus* may be attributed to significant difference in their constituents, like phenols, alkaloids and tannins [92], that might penetrates the cell membrane, combined with proteins of membranes and enzymes, loss of macromolecules from the interior of the cell, and eventually cell death [93, 94].

The expression levels of *afLJ*, *nor-1*, *afLR* and *pKsA* in the selected isolate treated with clove, peppermint, cinnamon and garlic oils were studied. The tested genes were down-regulated by the essential oils in a concentration dependent manner. The highest noticeable suppression of the synthetic and regulatory genes of aflatoxins was reported in response to cinnamon oil )0.0625 %). Similarly, the expression of *aflR*, *aflT*, *aflD*, *aflM*, and *aflP* were down-regulated by cinnamaldehyde, eugenol, and citral [60]. The expression of aflatoxin biosynthetic genes *ver-1*, *nor-1*, *pksA*, *omtA* and *aflR* was inhibited by eugenol and aflatoxin B1 production was inhibited by eugenol in the range of 15.07–98.0 % [95]. These results indicate that cinnamaldehyde and eugenol may be employed successfully as good candidate in controlling of fungal growth and subsequently contamination with aflatoxins of food, feed and agricultural commodities. The fungicidal action of essential oils was by destroying the structure and function of the cell wall and cell membrane, by changing the suitable external growth environment or cell physiological conditions for fungi and so the fungal cell lose the suitable environment for growth and metabolism. As well as, the antifungal effects of essential oils could be by blocking the biochemical cascade reactions, destroying the cellular signal pathways related to the growth, differentiation, reproduction, and metabolism of cells [95–98]. Similar results confirm the antiaflatoxigenic activity of cinnamon and spearmint essential oils in wheat grains [99]. The growth of aflatoxins producing *A. flavus* on peanut seeds was reduced upon addition of clove, cinnamon, thyme and peppermint [100]. The slight higher antifungal activity and antiaflatoxigenic potency of *A. flavus* under submerged than solid state fermentation conditions in response to the essential oils, could be due to the complexity of composition of food, pH, water activity, storage temperature, the presence of O_2,_ of the solid fermented cultures [101]. Since, the higher content of fat and / or proteins protects the microorganisms from the effects of essential oils, in addition to the relative accessibility to reach to the fungal spores in the lipid phase of food, so a relatively small portion remains free to act on the microorganisms in the aqueous phase, in addition to the reduced water activity of food of wheat grains that hinders the progress of essential oils towards microorganisms. As well as, the physical structure of food could make an extra barrier to limiting the antimicrobial effect of EO to reach the microorganisms [102].

In conclusion, contamination of wheat grains with aflatoxigenic fungi, due to the improper storage conditions and high humidity, was the main global threats to human and animal health. Thus, searching for novel strategies for controlling the growth of aflatoxigenic fungi on stored wheat grains was the challenge. *Aspergillus flavus* EFBL-MU12 PP087400, EFBL-MU23 PP087401 and EFBL-MU36 PP087403 isolates were the most potent aflatoxins producers on wheat grains, at incubation temperature 35°C, 16% moisture contents, initial pH 5.0, incubated for 14 days. Cinnamon oil had the highest fungicidal activity for growth and aflatoxins producing potency of *A. flavus* at 0.125%, in addition to the morphological aberrations to the fungal cell. The molecular expression of the aflatoxins biosynthetic genes *nor-1, afLR*, *pKsA* and *afLJ* genes by RT-qPCR were strongly reduced in response to treatment with cinnamon oil. Conclusively, cinnamon oils followed by peppermint oils displayed the most fungicidal activity for the growth and aflatoxins production by *A. flavus* grown on wheat grains.

## Supplementary Information

**Supplementary Material 1.** 

**Supplementary Material 2.** 

## Data Availability

The ITS sequences of *A. flavus* EFBL-MU12, EFBL-MU23, and EFBL-MU36 were deposited to Genbank with accession numbers PP087400 (https://www.ncbi.nlm.nih.gov/nuccore/PP087400), PP087401 (https://www.ncbi.nlm.nih.gov/nuccore/PP087401) and PP087403 (https://www.ncbi.nlm.nih.gov/nuccore/PP087403), respectively.
