# Supplementary material for: Potential fungicidal and antiaflatoxigenic effects of cinnamon essential oils on Aspergillus flavus inhabiting the stored wheat grains
Source: BMC Plant Biol. 2024 May 13;24:394. doi: 10.1186/s12870-024-05065-w (PMC11613666; doi:10.1186/s12870-024-05065-w)
Supplement: Supplementary file 1 — Supplementary Material 1. [file 12870_2024_5065_MOESM1_ESM.pptx]

## Slide 1
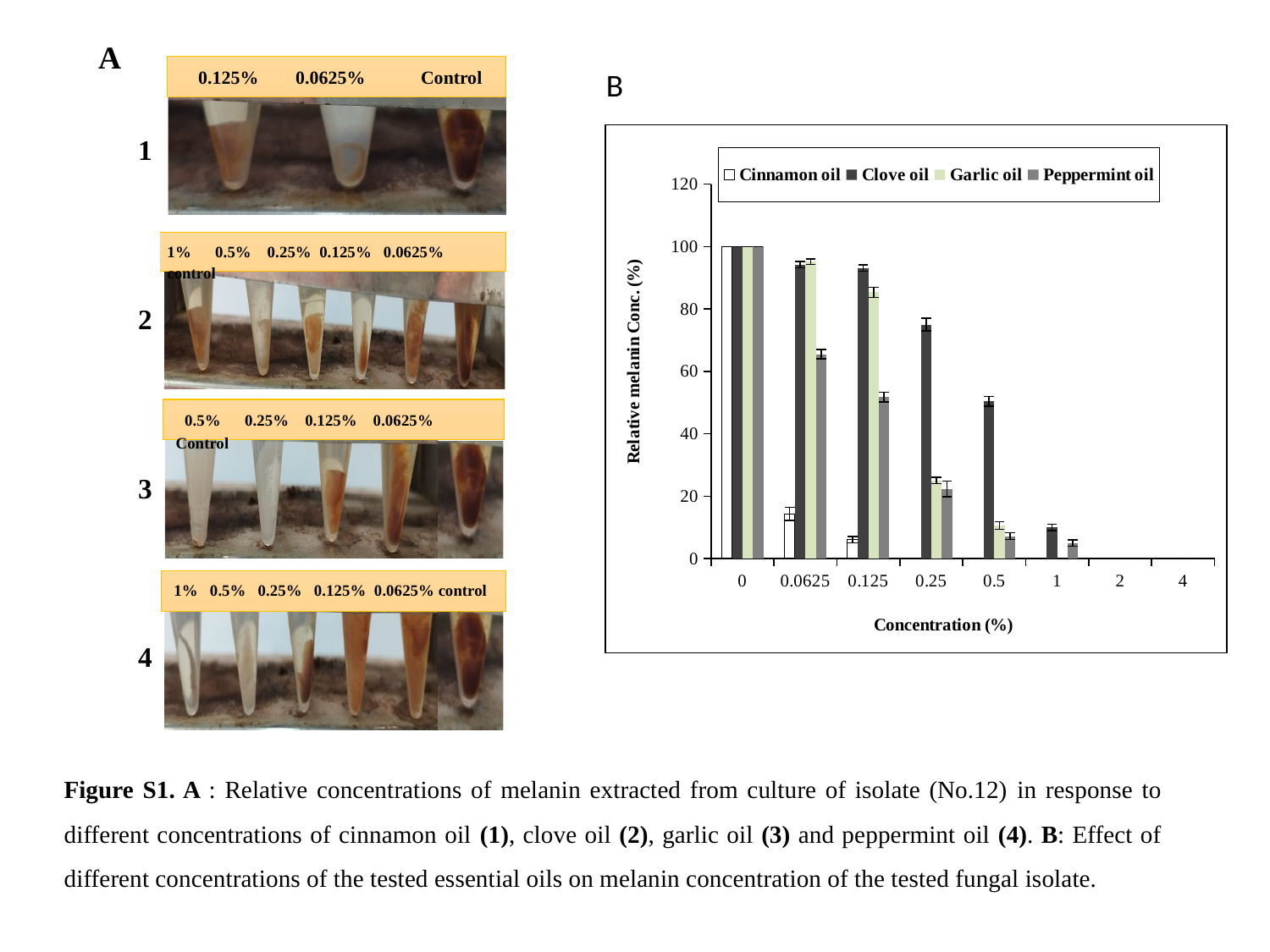

A
 0.125% 0.0625% Control
B
1
2
3
4
### Chart
| Category | | | | |
|---|---|---|---|---|
| 0 | 100.0 | 100.0 | 100.0 | 100.0 |
| 6.25E-2 | 14.3 | 94.2 | 95.1 | 65.5 |
| 0.125 | 6.0 | 93.1 | 85.3 | 51.7 |
| 0.25 | 0.0 | 75.0 | 25.1 | 22.3 |
| 0.5 | 0.0 | 50.4 | 10.6 | 7.2 |
| 1 | 0.0 | 10.0 | 0.0 | 5.0 |
| 2 | 0.0 | 0.0 | 0.0 | 0.0 |
| 4 | 0.0 | 0.0 | 0.0 | 0.0 |1% 0.5% 0.25% 0.125% 0.0625% control
 0.5% 0.25% 0.125% 0.0625% Control
1% 0.5% 0.25% 0.125% 0.0625% control
Figure S1. A : Relative concentrations of melanin extracted from culture of isolate (No.12) in response to different concentrations of cinnamon oil (1), clove oil (2), garlic oil (3) and peppermint oil (4). B: Effect of different concentrations of the tested essential oils on melanin concentration of the tested fungal isolate.

## Slide 2
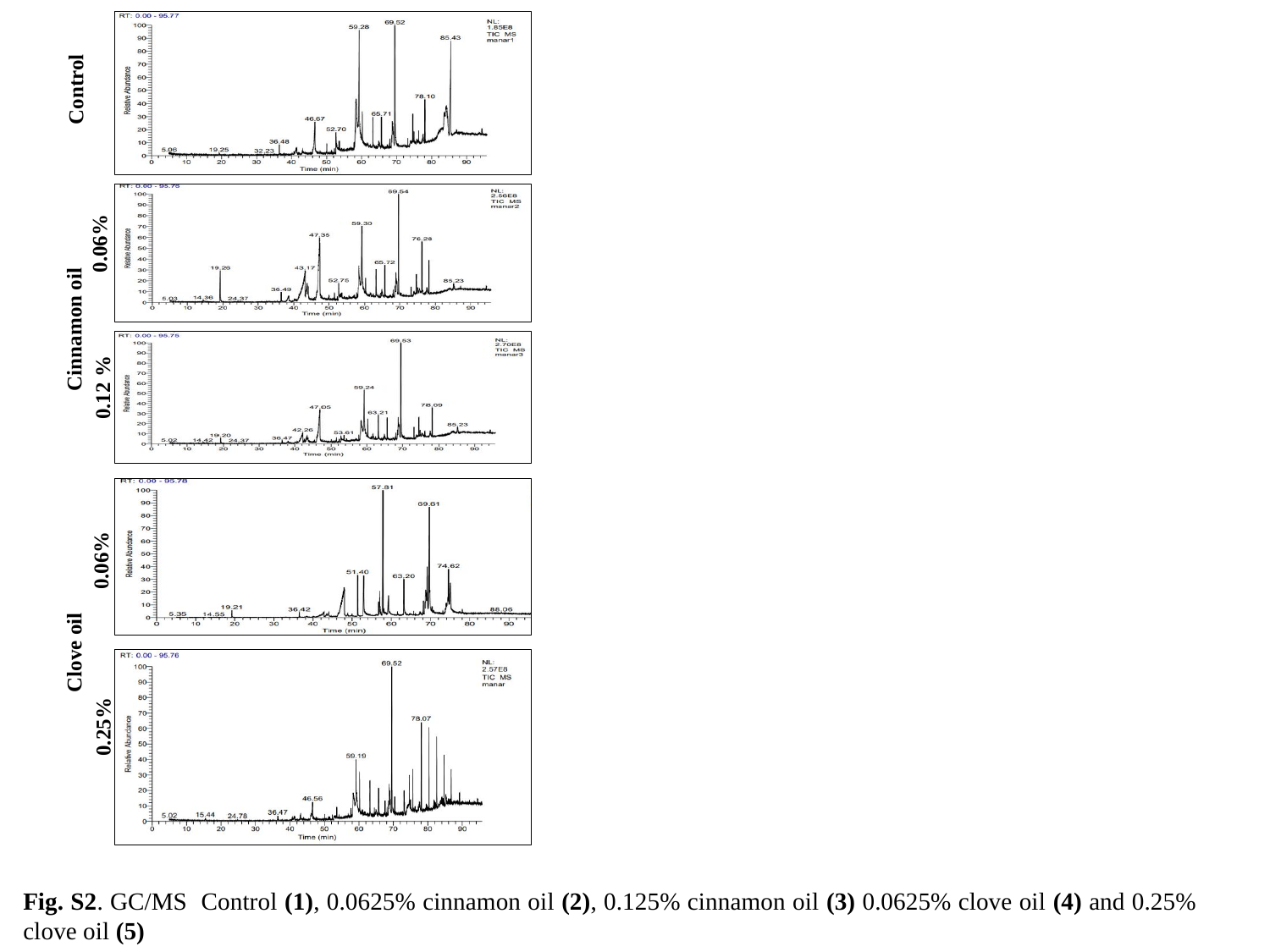

Control
0.06%
Cinnamon oil
0.12 %
0.06%
Clove oil
0.25%
Fig. S2. GC/MS Control (1), 0.0625% cinnamon oil (2), 0.125% cinnamon oil (3) 0.0625% clove oil (4) and 0.25% clove oil (5)
