# Supplementary material for: Potential fungicidal and antiaflatoxigenic effects of cinnamon essential oils on Aspergillus flavus inhabiting the stored wheat grains
Source: BMC Plant Biol. 2024 May 13;24:394. doi: 10.1186/s12870-024-05065-w (PMC11613666; doi:10.1186/s12870-024-05065-w)
Supplement: Supplementary file 2 — Supplementary Material 2. [file 12870_2024_5065_MOESM2_ESM.docx]

**Table S1. Aflatoxins production by the recovered fungal isolates from the stored wheat grains, by growing on Yeast Extract-Sucrose broth media.**

| **Sample No.** | **Fungal isolates** | **Isolate No.** | **Aflatoxins concentration (μg/L)** | | **Total amount of aflatoxins ( (μg/L)** |
| --- | --- | --- | --- | --- | --- |
|  |  |  | **B_1_** | **B_2_** |  |
| **1** | ***Aspergillus flavus*** | **1** | **-** | **42.57** | **42.57** |
|  | ***A. flavus*** | **2** | **-** | **54.69** | **54.69** |
|  | ***A .flavus*** | **3** | **-** | **-** | **0** |
|  | ***A .flavus*** | **4** | **-** | **-** | **0** |
|  | ***A .flavus*** | **5** | **-** | **66.01** | **66.01** |
| **2** | ***A .flavus*** | **6** | **-** | **18.65** | **18.65** |
|  | ***A .flavus*** | **7** | **-** | **53.07** | **53.07** |
| **3** | ***A .flavus*** | **8** | **-** | **35.44** | **35.44** |
| **4** | ***A .flavus*** | **9** | **-** | **29.44** | **29.44** |
| **5** | ***A. aflatoxiformans*** | **10** | **201.97** | **36.39** | **238.36** |
| **6** | ***A. flavus*** | **11** | **-** | **28.04** | **28.04** |
| **7** | ***A .flavus*** | **12** | **517.83** | **22.58** | **540.41** |
|  | ***A. aflatoxiformans*** | **13** | **125.57** | **17.5** | **143.07** |
| **8** | ***A .parasiticus*** | **14** | **-** | **40.98** | **40.98** |
|  | ***A. flavus*** | **15** | **-** | **-** | **0** |
| **9** | ***A. flavus*** | **16** | **-** | **30.03** | **30.03** |
| **10** | ***A. flavus*** | **17** | **-** | **30.48** | **30.48** |
| **11** | ***A. flavus*** | **18** | **-** | **40.47** | **40.47** |
| **12** | ***A. flavus*** | **19** | **-** | **79.86** | **79.86** |
|  | ***A. flavus*** | **20** | **-** | **-** | **0** |
|  | ***A. parasiticus*** | **21** | **-** | **-** | **0** |
|  | ***A. parasiticus*** | **22** | **-** | **68.51** | **68.51** |
| **13** | ***A. flavus*** | **23** | **404.23** | **10.42** | **414.65** |
|  | ***A .parasiticus*** | **24** | **-** | **-** | **0** |
| **14** | ***A. flavus*** | **25** | **-** | **-** | **0** |
|  | ***A. flavus*** | **26** | **-** | **57.77** | **57.77** |
| **15** | ***A. flavus*** | **27** | **-** | **-** | **0** |
|  | ***A. flavus*** | **28** | **-** | **24.56** | **24.56** |
|  | ***A. parasiticus*** | **29** | **-** | **33.11** | **33.11** |
| **16** | ***A. flavus*** | **30** | **-** | **50.77** | **50.77** |
|  | ***A. flavus*** | **31** | **-** | **36.39** | **36.39** |
|  | ***A. flavus*** | **32** | **-** | **40.32** | **40.32** |
| **17** | ***A. aflatoxiformans*** | **33** | **-** | **40.13** | **40.13** |
|  | ***A. flavus*** | **34** | **-** | **-** | **0** |
|  | ***A .flavus*** | **35** | **-** | **30.5** | **30.5** |
| **18** | ***A. flavus*** | **37** | **-** | **-** | **0** |
|  | ***A. flavus*** | **38** | **146.15** | **14.27** | **160.42** |
| **19** | ***A. parasiticus*** | **39** | **74.50** | **24.82** | **99.32** |
|  | ***A. flavus*** | **40** | **57.21** | **22.26** | **79.47** |
| **20** | ***A. flavus*** | **41** | **-** | **-** | **0** |
|  | ***A. flavus*** | **42** | **-** | **75.48** | **75.48** |
|  | ***A. flavus*** | **43** | **-** | **-** | **0** |
|  | ***A. flavus*** | **44** | **-** | **62.07** | **62.07** |
| **21** | ***A. flavus*** | **45** | **-** | **22.63** | **22.63** |
|  | ***A. flavus*** | **46** | **-** | **-** | **0** |
| **22** | ***A. flavus*** | **47** | **-** | **78.61** | **78.61** |
| **23** | ***A. flavus*** | **48** | **-** | **-** | **0** |
| **24** | ***A. flavus*** | **49** | **174.45** | **9.14** | **183.59** |
|  | ***A. flavus*** | **50** | **110.98** | **18.77** | **129.75** |
| **25** | ***A. parasiticus*** | **51** | **-** | **70.52** | **70.52** |
|  | ***A. flavus*** | **52** | **-** | **72.86** | **72.86** |
|  | ***A. flavus*** | **53** | **-** | **-** | **0** |
| **26** | ***A. flavus*** | **54** | **-** | **108.03** | **108.03** |
|  | ***A. flavus*** | **55** | **-** | **181.31** | **181.31** |
|  | ***A. parasiticus*** | **56** | **-** | **-** | **0** |
|  | ***A. parasiticus*** | **57** | **-** | **-** | **0** |
| **27** | ***A. flavus*** | **58** | **-** | **158.46** | **158.46** |
|  | ***A. flavus*** | **59** | **-** | **151.8** | **151.8** |
|  | ***A. flavus*** | **60** | **-** | **90.86** | **90.86** |
| **28** | ***A. flavus*** | **61** | **-** | **34.19** | **34.19** |
|  | ***A. flavus*** | **62** | **-** | **-** | **0** |
|  | ***A. parasiticus*** | **63** | **10.21** | **9.26** | **19.47** |
|  | ***A. parasiticus*** | **64** | **15.21** | **4.29** | **19.5** |
| **29** | ***A. aflatoxiformans*** | **65** | **99.59** | **12.27** | **111.86** |
|  | ***A. aflatoxiformans*** | **66** | **74.51** | **24.82** | **99.33** |
|  | ***A. flavus*** | **67** | **-** | **-** | **0** |
| **30** | ***A. flavus*** | **68** | **-** | **13.37** | **13.37** |
|  | ***A. flavus*** | **69** | **-** | **-** | **0** |
|  | ***A. flavus*** | **70** | **-** | **23.8** | **23.8** |

**The aflatoxins productivity by the fungal isolates collected from the different stored wheat grains were assessed by growing on Yeast Extract-Sucrose broth medium. Seventy fungal isolates belongs to the *A. flavus* group were isolated from 30 samples of wheat grains collected from different local markets in Egypt. After incubation, the cultures were filtrated and the aflatoxins were extracted, and preliminary assessed by the TLC, as described in Materials and Methods.**

| Variables | Responses | | | | | |
| --- | --- | --- | --- | --- | --- | --- |
|  | Aflatoxin B_1_ (μg/kg) | | | Aflatoxin B_2_ (μg/kg) | | |
| Source | Sum of squares | *F-value* | *p-value* | Sum of squares | *F-value* | *p-value* |
| Regression | 96443.8 | 53.06 | 0.000 | 1924.41 | 26.22 | 0.000 |
| Linear | 35561.6 | 68.47 | 0.000 | 429.56 | 20.49 | 0.000 |
| Temperature | 4750.0 | 36.58 | 0.000 | 113.58 | 21.67 | 0.000 |
| Moisture content (MC) | 13492.9 | 103.92 | 0.000 | 52.90 | 10.09 | 0.006 |
| pH | 3189.7 | 24.57 | 0.000 | 34.49 | 6.58 | 0.021 |
| Incubation time | 14129.0 | 108.82 | 0.000 | 228.60 | 43.61 | 0.000 |
| Square | 56398.1 | 108.59 | 0.000 | 1358.96 | 64.81 | 0.000 |
| Temp*Temp | 9199.1 | 130.74 | 0.000 | 99.36 | 47.07 | 0.000 |
| MC*MC | 5865.9 | 84.72 | 0.000 | 441.51 | 117.82 | 0.000 |
| pH*pH | 18696.6 | 177.86 | 0.000 | 472.04 | 106.09 | 0.000 |
| Time*Time | 22636.6 | 174.34 | 0.000 | 346.05 | 66.01 | 0.000 |
| Interaction | 4484.1 | 5.76 | 0.002 | 135.88 | 4.32 | 0.009 |
| Temp*MC | 443.9 | 3.42 | 0.083 | 11.21 | 2.14 | 0.163 |
| Temp*pH | 21.5 | 0.17 | 0.690 | 0.59 | 0.11 | 0.742 |
| Temp*Time | 275.4 | 2.12 | 0.165 | 0.05 | 0.01 | 0.924 |
| MC*pH | 1006.5 | 7.75 | 0.013 | 36.33 | 6.93 | 0.018 |
| MC*Time | 67.0 | 0.52 | 0.483 | 10.84 | 2.07 | 0.170 |
| pH*Time | 2669.8 | 20.56 | 0.000 | 76.87 | 14.66 | 0.001 |
| R-Square (%) | 97.89 | | | 95.82 | | |

**Table S2: Analysis of variance (ANOVA) of the CCD experiment for the calculated responses of aflatoxins production by A. flavus**

**The Analysis of variance (ANOVA) parameters of aflatoxins production by *A. flavus* (isolate #12) grown on wheat grains under solid state fermentation, were nutritionally optimized by the Response Surface Methodology with Central Composite Design (CCD) (CCD). The selected parameters were firstly screened from the Plackett-Burman Design, and optimized by the CCD, including initial moisture contents, incubation time, incubation temperature and initial pH of the moistening solution. After incubation of the cultures, aflatoxins were extracted from the cultures and quantified by TLC and HPLC as described in Materials and Methods. The significance of the tested parameters as Sum of Squares, F-value and *p*-values were calculated as revealed from ANOVA analysis.**

| **Conc. of essential oil (%)** | **Cinnamon oil** | | **Clove oil** | | **Garlic oil** | | **Peppermint oil** | |
| --- | --- | --- | --- | --- | --- | --- | --- | --- |
|  | **Colony diameter (cm)** | **Mycelial growth inhibition(%)** | **Colony diameter (cm)** | **Mycelial growth inhibition(%)** | **Colony diameter (cm)** | **Mycelial growth inhibition(%)** | **Colony diameter (cm)** | **Mycelial growth inhibition(%)** |
| **Control** | 8.5^a^±0.5 | 0^c^±0.0 | 8.5^a^±0.5 | 0^e^±0.0 | 8.5^a^±0.5 | 0^e^±0.0 | 8.5^a^±0.5 | 0^f^±0.0 |
| **0.0625%** | 1.2^b^±0.3 | 86.3^b^±2.8 | 7.8^b^±0.3 | 13.7^d^±2.8 | 7.7^b^±0.2 | 14.1^d^±2.3 | 6.5^b^±0.2 | 27.0^e^±2.3 |
| **0.125%** | 0.5^c^±0.1 | 94.4^a^±1.2 | 7.7^bc^±0.3 | 14.4^d^±2.9 | 6.5^c^±0.3 | 27.8^c^±2.9 | 5.4^c^±0.06 | 39.6^d^±0.6 |
| **0.25%** | - | - | 7.1^cd^±0.2 | 20.7^c^±1.7 | 4.5^d^±0.5 | 50^b^±5.6 | 2.7^d^±0.2 | 69.6^c^±2.3 |
| **0.5%** | - | - | 6.6^d^±0.4 | 27.03^b^±4.5 | 2.2^e^±0.2 | 75.6^a^±2.3 | 1.4^e^±0.06 | 84.0^b^±0.6 |
| **1%** | - | - | 2.2^e^±0.3 | 75.2^a^±2.8 | - | - | 0.8^f^±0.3 | 90.7^a^±3.2 |
| **2%** | - | - | - | - | - | - | - | - |
| **4%** | - | - | - | - | - | - | - | - |
| **LSD** | 0.66 | 3.47 | 0.58 | 5.01 | 0.62 | 5.27 | 0.47 | 3.38 |

**Table S3: Effect of different concentrations of the tested essential oils on the radial growth of A. flavus (isolate # 12).**

**The antifungal activity of the different oils on the growth of the most aflatoxins producing isolate of *A. flavus* (#12) were assessed by growing on Yeast Extract-Sucrose Agar media, amended with different concentration of each essential oil. After incubation of the cultures, the diameters of the colony and mycelial growth rate were estimated as described in Materials and Methods.**

**Each value represents the mean of 3 replicates (Mean ± SD). The same letters in each column represents insignificant difference where LSD at *p* ≤ 0.05**

**Table S4: Effect of different concentrations of the tested essential oils on pigment production by A. flavus (isolate # 12)**

| **Conc. of essential oil (%)** | **Cinnamon oil** | **Clove oil** | **Garlic oil** | **Pepper mint oil** |
| --- | --- | --- | --- | --- |
|  | **Relative melanin concentration (%)** | | | |
| **Control** | 100^a^±0.0 | 100^a^±0.0 | 100^a^±0.0 | 100^a^±0.0 |
| **0.0625%** | 14.3^b^±2.1 | 94.2^b^±0.98 | 95.1^b^±0.9 | 65.5^b^±1.6 |
| **0.125%** | 6^c^±1 | 93.1^b^±1 | 85.3^c^±1.6 | 51.7^c^±1.6 |
| **0.25%** | - | 75^c^±2 | 25.1^d^±1.03 | 22.3^d^±2.5 |
| **0.5%** | - | 50.4^d^±1.5 | 10.6^e^±1.3 | 7.2^e^±1.04 |
| **1%** | - | 10^e^±1 | - | 5^e^±1 |
| **2%** | - | - | - | - |
| **4%** | - | - | - | - |
| **LSD** | 2.66 | 2.21 | 2.004 | 2.64 |

**The effect of different essential oils on melanin pigmentation of A. flavus (isolate # 12) were assessed by growing the fungus on Yeast-extract broth media, amended with different concentrations of each oil. After incubation, the fungal mycelial biomass was collected by filtration, and melanin was extracted, and quantified as described in Materials and Methods. The values were represented by the means of 3 replicates and standard deviation. The same letters in each column represents the insignificant difference where LSD at p ≤ 0.05.**
